# Seeing the unseen: A novel approach to extract latent plant root traits from digital images

**DOI:** 10.1016/j.plaphe.2025.100088

**Published:** 2025-07-09

**Authors:** Mirza Shoaib, Adam M. Dimech, Simone J. Rochfort, Christopher Topp, Matthew J. Hayden, Surya Kant

**Affiliations:** a Agriculture Victoria, Grains Innovation Park, 110 Natimuk Road, Horsham, Victoria, 3400, Australia; b School of Applied Systems Biology, La Trobe University, Bundoora, Victoria, 3083, Australia; c Agriculture Victoria, AgriBio, Centre for AgriBioscience, 5 Ring Road, Bundoora, Victoria, 3083, Australia; d Donald Danforth Plant Science Center, Saint Louis, Missouri, USA; e Department of Ecological, Plant and Animal Science, School of Agriculture, Biomedicine & Environment, La Trobe University, Bundoora, Victoria, 3083, Australia

**Keywords:** Algorithmic Root Traits (ART), Root phenotyping, Drought tolerance, Wheat, Latent trait, Machine learning, Root image analysis

## Abstract

A novel approach, the Algorithmic Root Trait (ART) extraction method, identifies and quantifies computationally-derived plant root traits, revealing latent patterns related to dense root clusters in digital images. Using an ensemble of multiple unsupervised machine learning algorithms and a custom algorithm, 27 ARTs were extracted reflecting dense root cluster size and spatial location. These ARTs were then used independently and in combination with Traditional Root Traits (TRTs) to classify wheat genotypes differing in drought tolerance.

ART-based models outperformed TRT-only models in drought classification (e.g., 96.3 ​% vs. 85.6 ​% accuracy). Combining ARTs and TRTs further improved accuracy to 97.4 ​%. Notably, 4 selected ARTs matched the performance of all 23 TRTs, offering 5.8 ​× ​higher information density (0.213 vs. 0.037 accuracy/feature). This superiority reflects the ability of ARTs to capture richer, more complex architectural information, evidenced by higher internal variability (35.59 ​± ​11.41 vs. 28.91 ​± ​14.28 for TRTs) and distinct data structures in multivariate analyses; PERMANOVA confirmed that ARTs and TRTs provide complementary insights.

Validated through experiments in controlled environments and field conditions with wheat drought-tolerant and susceptible genotypes, ART offers a scalable, customisable toolset for high-throughput phenotyping of plant roots. By bridging conventional, visually derived traits with autonomous computational analyses, this method broadens root phenotyping pipelines and underscores the value of harnessing sensor data that transcends human perception. ART thus emerges as a promising framework for revealing hidden features in plant imaging, with broader applications across plant science to deepen our understanding of crop adaptation and resilience.

## 1 Introduction

While automated data analysis tools effectively extract valuable root traits [[1], [2], [3], [4], [5]], these typically rely on predefined geometric or morphological characteristics derived from human visual perception. Consequently, subtle or complex patterns within image data, which do not align neatly with established definitions, may remain undetected [[6], [7], [8], [9], [10]]. The identification of latent, complex image-derived traits offers considerable potential for advancing plant phenotyping, particularly for roots, which are inherently concealed and interact dynamically with their environment [[11], [12], [13]]. In this manuscript, “latent traits” are defined as algorithmically identified, interpretable features—such as root cluster location, size, and density—derived from pixel distributions rather than human visual assessments or abstract neural network representations.

A comprehensive understanding of plant roots, essential for productivity and climate adaptation, is required to facilitate the breeding of climate-resilient crops [[14], [15], [16]]. Critical root attributes such as architecture, structure, anatomy, plasticity, exudation, microbial interactions, and hydraulics significantly influence plant performance [[17], [18], [19], [20], [21], [22], [23]]. Among these, architectural and structural traits have been primary research targets due to their measurability through phenotyping techniques like shovelomics, rhizotrons, minirhizotrons, and soil coring [[24], [25], [26], [27]]. Software tools such as RhizoVision, WinRHIZO, RootNav, DIRT, and ImageJ typically extract these traits, termed Traditional Root Traits (TRTs) herein, from segmented root images [[1], [2], [3], [4], [5]].

Despite their utility, TRTs may inadequately capture root complexity especially under stress conditions like drought [28]. Roots navigate unique environmental challenges—darkness, variable moisture, mechanical impedance—distinct from aerial tissues, complicating their phenotypic characterisation [[11], [12], [13]].

Alternative approaches have emerged to overcome these limitations. Berrigan et al. [29] adapted SLEAP, a deep-learning animal motion capture system, for root landmark detection without segmentation. Ubbens et al. [30] proposed Latent Space Phenotyping using convolutional neural networks for automatic trait detection from images. Peeples et al. [31] employed Earth Mover's Distance for insights into root system architecture beyond conventional metrics, while Li et al. [32] introduced persistent-homology-based topological methods to quantify morphological variations.

Building on these advances, we introduce the Algorithmic Root Trait (ART) extraction method (see Table S1 for a comparative overview with existing analytical methods). ART systematically integrates multiple unsupervised machine learning (ML) algorithms with a custom-developed algorithm (hereafter, the "Custom" algorithm) to identify and characterise dense root clusters. The novelty of ART lies in harnessing the complementary strengths of these algorithms, uncovering spatial patterns and architectural features often missed by traditional methods.

Unlike methods emphasising global distribution comparisons [31] or abstract topological metrics [32], ART provides directly interpretable localised geometric and density-based traits (e.g., cluster size, centroid coordinates). Additionally, ART autonomously detects emergent root clustering patterns without relying on predefined assumptions or supervised landmark detection approaches [29].

ART's inherent flexibility enables seamless integration with TRTs, facilitating comprehensive analyses of root architecture. Leveraging an algorithmically unconstrained approach, ART extracts latent traits from digital RGB sensor data, significantly enhancing the discovery of nuanced, physiologically relevant traits critical for understanding plant resilience.

To demonstrate ART's practical value, experiments under controlled environment and field conditions were conducted to classify wheat genotypes based on drought tolerance. Wheat, central to global food security [33], faces increasing drought stress, predicted to surpass pathogen threats in its impact on crop productivity [34]. Root density, reflecting plant resource allocation under drought, varies with soil depth and between tolerant and susceptible genotypes [[35], [36], [37]]. Given environmental influences and subjective assessments, accurately identifying dense root areas is challenging.

ART's unsupervised clustering methods were applied to objectively analyse RGB sensor-derived data, identifying dense root clusters to generate informative traits. Integrating these ART-derived traits with TRTs within a supervised ML pipeline improved genotype classification accuracy. Further, combining ARTs with TRTs substantially enhanced predictive performance. Evaluations across controlled environment and variable field conditions confirmed ART's robust performance, underscoring its potential applicability across diverse research and agricultural contexts.

## 2 Materials and methods

### 2.1 Experiment conditions and genotypes

A glasshouse rhizotron study was conducted to compare three previously characterised drought-tolerant (DT_1, DT_2, DT_3) and three drought-susceptible (DS_1, DS_2, DS_3) wheat (*Triticum aestivum* L.) genotypes (Table S2) [[38], [39], [40], [41]]. The experiment utilised 48 rhizotrons (594 ​cm height ​× ​42 ​cm width ​× ​6 ​cm depth; volume: 6.5 ​L) filled with fertilised coco peat (Table S3), arranged in a completely randomised design with four replicates per genotype per treatment (control and drought). The drought treatment was maintained at 30 ​% field capacity (FC), while the control was maintained at 60 ​% FC. Rhizotrons were angled at 45° [38] and wrapped with opaque material to exclude light and mitigate temperature fluctuations. Water was applied by weight to maintain FC.

Three seeds were sown per rhizotron and thinned to a single seedling post-germination. A pilot study determined optimal seed orientation (brush up, crease facing rhizotron wall) for consistent root trait expression (Fig. S1) [39]. Key physiological traits (leaf relative water content, stomatal conductance, tiller number) were recorded at 45 and 49 days after sowing (DAS) to validate genotype drought responses (see ‘Drought Tolerance Characterisation' in Result section).

A complementary field experiment was conducted at the research farm of Agriculture Victoria, Horsham (36°43'57″S, 142°06'01″E), planted two contrasting genotypes (DT_1 and DS_3) in a randomised complete block design. Three replicate plots (5 ​m ​× ​1 ​m) per genotype (6 plots total) were established, with five rows per plot.

### 2.2 Imaging setup

A custom glasshouse imaging setup captured rhizotron images (Fig. S2). It featured a blackout curtain, a fixed camera-rhizotron frame, matt black paint, a light blue background (HEX: #0089b6), and four diffused studio lights. Images (n ​= ​960) were taken at 20 time-points (day 5–49) with a Sony camera a7R using Sony FE 35 ​mm F1.4 ​GM Lens (Sony Corporation, Tokyo, Japan).

For imaging at field experiment, four weeks after sowing, three minirhizotron tubes (65 ​mm diameter, 1.2-m long) were installed at 45° within each plot (18 tubes total). These minirhizotron tubes were scanned using an In-Situ Root Imager ICI-600 (CID-Bio-Science, Camas, Washington, United States). Each minirhizotron was scanned three times from top to bottom, resulting in 54 images per imaging day (3 scans ​× ​18 tubes). Seven time-series scans were captured at specific intervals: 147, 151, 157, 163, 170, 177, and 184 DAS, producing a total of 378 images. The field and glasshouse experimental setups are depicted in Fig. S3.

### 2.3 Image processing pipeline

Python 3.10.2 [40] and R-4.3.2 [41] were used for image preprocessing and analysis. Glasshouse images underwent automated preprocessing: QR code decoding (pyzbar library) for renaming and sample tracking, date suffixing, EXIF-based rotation, and cropping into shoot and root regions (3410 x 5075 pixels for roots) based on fixed coordinates. Field minirhizotron images (three per tube) were merged and oriented, yielding 126 composite images from the original 378.

Images were segmented using RootPainter [42]. To handle distinct imaging environments (glasshouse vs. field), two separate segmentation models were developed. The model was first trained for glasshouse images. This optimised glasshouse model then served as a pre-trained base for fine-tuning with field-specific annotations, reducing training time and adapting to differences in growing media and root morphology. For both datasets, representative images were manually annotated, and models were trained iteratively until the Dice score neared 1 (Fig. S4). The best-performing glasshouse model segmented all glasshouse images, and the fine-tuned field-specific model segmented all field images.

### 2.4 Traditional Root Traits (TRTs) extraction

**Fig. 1:**
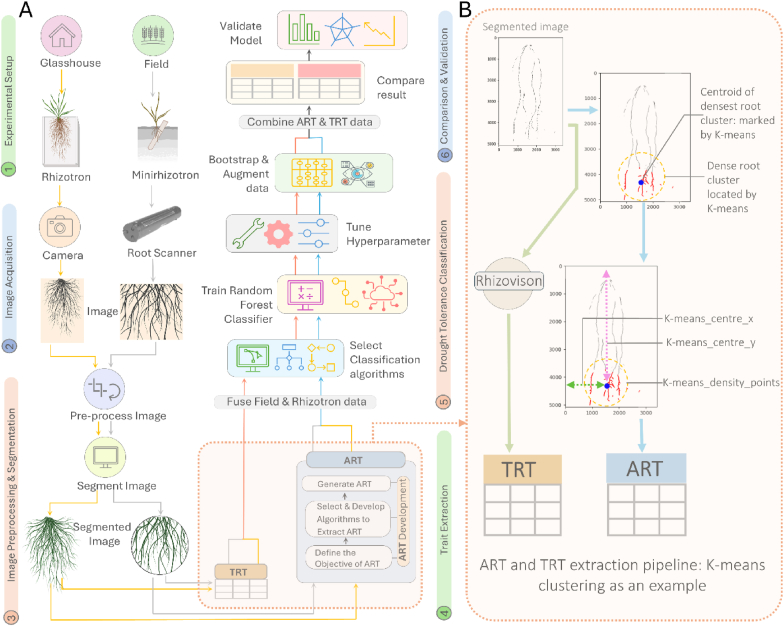
Experimental setup and workflow. (A) Schematic workflow of ART and TRT extraction and drought tolerance classification. (1) Experimental setup (2) Image Acquisition: Custom imaging setup for glasshouse and C-600 root imager for field. (3) Image preprocessing and segmentation. (4) ART and TRT extraction. (5) Drought tolerance classification with the Random Forest algorithm. (6) Classification result comparison and validation. (B) ART and TRT extraction pipeline: TRT extraction using Rhizovision and TRT extraction using K-means Clustering Algorithm.

TRTs were extracted from segmented binary images (glasshouse and field) using RhizoVision Explorer [10] (Fig. 1B) with the "broken roots" setting (Table S4) for consistency. This yielded 23 TRTs (listed in Table S5). Field (n ​= ​126) and glasshouse (n ​= ​960) TRT datasets were merged (total n ​= ​1086).

### 2.5 Defining Algorithmic Root Trait (ART)

TRTs typically quantify pre-defined global or skeleton-based geometric/morphological features [[1], [2], [3],^5^]. While valuable, they may not fully capture complex spatial organisation and density variations within the root system. The Algorithmic Root Trait (ART) extraction method, introduced here, employs an ensemble of unsupervised ML algorithms and the Custom algorithm (Table 1) applied post-segmentation.

**Table 1:** List of unsupervised ML and custom algorithms used in ART extraction.

Algorithm Category	Algorithm Name	Specific Function	Cluster Shape	Reference
Partitioning	Gaussian Mixture Models (GMM)	Soft partitioning	Globular/spherical	Pedregosa et al. [47]
	K-means	Hard partitioning	Globular/spherical	Pedregosa et al. [47]
Density-based	Density-Based Spatial Clustering of Applications with Noise (DBSCAN)	Density-based clustering	Elongated/irregular	Pedregosa et al. [47] and Ester et al. [48]
	Mean-shift	Mode-seeking clustering	Elongated/irregular	Pedregosa et al. [47] and Comaniciu and Meer [49]
	Ordering Points to Identify Cluster Structure (OPTICS)	Density-based clustering (extension of DBSCAN)	Elongated/irregular	Pedregosa et al. [47] and Ankerst et al. [50]
Hierarchical	Hierarchical Density-Based Spatial Clustering of Applications with Noise (HDBSCAN)	Hierarchical density-based clustering	Elongated/irregular	McInnes et al. [51]
Graph-based	Fuzzy C-means (FCM)	Fuzzy clustering	Adaptable to different shapes	Bezdek et al. [52]
Superpixel Segmentation	Simple linear iterative clustering (SLIC)	Superpixel generation based on colour and spatial similarity	Irregular	Achanta et al. [53]
Custom	Custom	Density estimation and selection of the largest cluster	Density-based	Custom

ARTs are designed to uncover structural and spatial patterns by analysing segmented root pixel distributions, focusing on identifying and quantifying characteristics of algorithmically “discovered dense root clusters”. In this context, "latent" refers to cluster characteristics (precise location, boundaries, density) that emerge from data-driven algorithmic analysis rather than being predefined by human visual rules. While the extracted metrics (size, location) are interpretable, the discovery and delineation of clusters themselves are entirely algorithmic.

The novelty of ART lies in its systematic, multi-algorithmic approach to identify dense root clusters and extract interpretable traits: cluster size via [AlgorithmName]_density_points and spatial coordinates via [AlgorithmName]_centre_x/y (Table S5). Unlike methods emphasising global distributions, abstract topological metrics, or supervised landmark detection (Table S1), ART provides localised, interpretable traits from autonomously detected patterns. This approach offers a distinct perspective on root architecture, complementary to global TRT metrics (Fig. 3), as demonstrated by correlation and feature importance analyses (Figs. S5-S8, S14).

### 2.6 ART extraction

ARTs were extracted from binary segmented images using nine algorithms (eight established unsupervised ML methods plus one custom procedure; Table 1) to identify and characterise the largest dense root cluster in each image. The clustering approach was selected for two key reasons:

*Biological rationale:* Dense root clusters represent adaptive strategies where plants concentrate biomass in resource-rich zones to optimise water acquisition under drought [[35], [36], [37],^43^]. This spatial organisation reflects phenotypic plasticity consistent with established drought adaptation mechanisms, including strategic positioning for enhanced water uptake efficiency [[44], [45], [46]].

*Methodological rationale:* Clustering algorithms directly quantify biologically meaningful spatial patterns—cluster size (biomass concentration), centroid coordinates (spatial positioning), and density distributions—that correspond to functional root architecture components known to influence drought tolerance [43,46]. The ensemble approach captures diverse clustering morphologies, from compact aggregations to elongated formations (Table 1), reflecting the architectural diversity of adaptive root responses.

After feasibility screening, algorithms representing diverse clustering approaches were selected: DBSCAN (epsilon ​= ​5, min_samples ​= ​1 0), Gaussian Mixture Models (GMM; n_components ​= ​2), K-means (n_clusters ​= ​5), HDBSCAN (min_cluster_size ​= ​10), MeanShift (quantile ​= ​0.1), OPTICS (min_samples ​= ​10), SLIC (n_segments ​= ​50), and Fuzzy C-means (FCM). Parameters (Table 1) were optimised in preliminary experiments.

The Custom algorithm identifies dense regions as follows: (1) All root pixels (black pixels) are identified. (2) If ​> ​200 root pixels exist, the 200 most locally dense (pixels surrounded by other root pixels) are selected. (3) A Gaussian kernel density estimate (KDE) weights these selected pixels. (4) Density-weighted coordinates are clustered using K-means (up to 5 clusters, depending on point availability). (5) The largest cluster (by point count) is chosen. (6) Traits extracted are Density_points (cluster size), Density_centre_x, and Density_centre_y (cluster geometric centre).

Each of the nine algorithms extracts three traits from its identified largest cluster: [AlgorithmName]_density_points (size), [AlgorithmName]_centre_x (horizontal position), and [AlgorithmName]_centre_y (vertical position), yielding 27 ARTs (Table S5). These are maintained as distinct features because each algorithm applies different mathematical principles to identify cluster boundaries from the same image data. For example, K-means partitions pixels into globular clusters while HDBSCAN identifies density-connected regions of arbitrary shape, resulting in different cluster definitions and centroid locations from identical inputs (Table 1, Fig. 3). Algorithm-specific naming (e.g., FCM_centre_x vs. DBSCAN_centre_x) preserves information about which computational approach identified each cluster, enabling determination of the most biologically informative clustering perspectives. This methodological diversity ensures comprehensive characterisation of root spatial organisation patterns. All code and data are available for download at https://github.com/shoaibms/ART.

### 2.7 Comparative dataset analysis and drought phenotyping

#### 2.7.1 Comparing TRT, ART and combined datasets

Internal variability (mean Euclidean distance), correlation matrices, Euclidean Distance Matrix Heatmaps, Principal Component Analysis (PCA), t-distributed Stochastic Neighbor Embedding (t-SNE), and Multidimensional Scaling (MDS) were used to compare ART, TRT, and combined datasets. PERMANOVA was used to test statistical differences between ART and TRT datasets.

#### 2.7.2 Drought response characterisation

To validate genotype drought responses, key physiological indicators were measured. Leaf Relative Water Content (RWC) was determined at 45 DAS using ∼100 ​mg samples from young fully expanded leaves. Fresh weight was recorded, followed by overnight hydration in deionised water at 4 ​°C for turgid weight, and oven-drying at 80 ​°C for 48 ​h for dry weight. RWC was calculated as [(FW-DW)/(TW-DW)] ​× ​100 ​% [54]. Stomatal conductance (g_s) was measured on the adaxial surface of the second fully expanded leaf using a portable photosynthesis system (LCpro-SD) between 10:00am-2:30pm to minimise diurnal effects. Measurements were taken at 45 DAS (g_s_1) and 49 DAS (g_s_2) under standardised conditions (PAR 1500 ​μmol ​m^−2^ ​s^−1^, ambient CO_2_). Tiller number was counted at 45 DAS. Effect sizes of these traits were analysed to support their use in genotype classification.

##### 2.7.2.1 Calculation of selected Trait's effects

Effect sizes (Cliff'‘s Delta) between tolerant and susceptible groups were assessed for g_s_1, g_s_2, RWC, and Tiller_no. Normality (Shapiro-Wilk) determined ANOVA (Tukey's HSD for normal RWC) or Kruskal-Wallis (Dunn's test for non-normal g_s_1, g_s_2, Tiller_no).

##### 2.7.2.2 Genotype ranking methodology

To quantitatively assess drought tolerance, genotypes were ranked based on their physiological responses using three complementary approaches. This methodology involved calculating two primary drought response metrics for each genotype (*i*) and physiological trait (*j*), from which three final ranking scores were derived.

**Variable Definitions:**

*i* ​= ​genotype index (from 1 to 6)

*j* ​= ​physiological trait index (g_s_1, g_s_2, RWC, tiller number)

*w*_*j*_ ​= ​trait weight (2 for stomatal conductance traits, 1 for RWC and tiller number)

**Primary Drought Response Metrics:**

To ensure that both metrics were robust to potential outliers in the data, 10 ​% trimmed means were used for all calculations.

1. Robust Difference (RD):

(1) RD_ij_ ​= ​∣M_ij,control_−M_ij,drought_∣

where M_ij,control_−M_ij,drought_ are the 10 ​% trimmed means for trait *j* of genotype *i* under control and drought conditions, respectively.

2. Robust Stress Susceptibility Index:

The Stress Susceptibility Index [55] quantifies the relative reduction in performance under stress conditions. A robust version was calculated using trimmed means to maintain consistency with the RD metric:

(2) $${SSI}_{ij}=1-\left(\frac{M_{ij,drought}}{M_{ij,control}}\right)$$

**Final Ranking Scores**.

For each metric (RD and SSI), genotypes are ranked from 1 (most tolerant) to 6 (most susceptible) for each of the four traits. The following weighted ranking scores are calculated:

3. RANK_1_ (Robust Difference-based Score):

(3) $${RANK}_{1,i}=\sum_{j}\left[Rank\left(\;{RD}_{ij}\right)\times w_{j}\right]$$

4. RANK_2_ (SSI-based Score):

(4) $${RANK}_{2,i}=\sum_{j}\left[Rank\left(\;{SSI}_{ij}\right)\times w_{j}\right]$$

5. RANK3 (SSI-based Score):

(5) $${RANK}_{3,i}={RANK}_{1,i}+{RANK}_{2,i}$$

Note: The summation ∑_*j*_ runs over all four traits. Lower RANK scores indicate higher drought tolerance.

##### 2.7.2.3 Clustering approach

Five unsupervised clustering algorithms (K-means, Agglomerative, Gaussian Mixture, Spectral, and Birch) classified wheat genotypes into “Tolerant” (T ​= ​1) and “Susceptible” (S ​= ​0) groups under both watering treatments (T0 and T1) using physiological traits (g_s_1, g_s_2, RWC, and tiller number). Data were treatment-segregated and standardised using StandardScaler before generating two clusters per algorithm. Cluster quality was assessed using internal metrics (Silhouette Score, Dunn Index, Calinski-Harabasz Index, and Davies-Bouldin Index) to evaluate separation, and external metrics (Adjusted Rand Index, Normalised Mutual Information, and Fowlkes-Mallows Score) to compare algorithmic assignments against known drought tolerance classifications.

#### 2.7.3 Classification framework

##### 2.7.3.1 Algorithm selection and evaluation

Eight classifiers (AdaBoost, CatBoost, Gradient Boosting, K-Nearest Neighbors, LightGBM, Logistic Regression, Random Forest and Support Vector Machine (SVM)) were evaluated for drought tolerance classification using ART and TRT datasets. Multiple metrics were evaluated including accuracy, precision, recall, F1 score, ROC AUC, and specificity, with priority given to precision and ROC AUC.

##### 2.7.3.2 Development and robust validation of classification models

A multi-stage validation strategy ensured robust evaluation and mitigated overfitting (Fig. S11):

• An independent validation set (n ​= ​218: 36 field, 182 glasshouse samples) was set aside at the beginning of the study and kept completely separate from all model development processes to provide an unbiased assessment for final model validation.
• Augmented Development Dataset: Remaining original samples (n ​= ​868) were augmented to create a development dataset (n ​= ​1736). Augmentation involved bootstrapping original samples with replacement and adding controlled Gaussian noise (σ ​= ​0.02) to numerical features; thus, the development dataset comprised both original and these newly generated bootstrapped/noised samples.
• Internal Split: This development dataset was split (stratified by drought tolerance) into an internal training set (80 ​%, n ​= ​1388) and an internal test set (20 ​%, n ​= ​348). No data leakage occurred between development and independent validation sets.

The Random Forest classifier was built using a pipeline comprising three sequential stages: (1) data standardisation with StandardScaler, (2) feature selection using SelectFromModel with a Random Forest estimator, and (3) final classification with the main Random Forest model.

Hyperparameter tuning was conducted exclusively on the internal training set (n ​= ​1388) using Grid Search CV with 10-fold cross-validation, optimising for precision. This cross-validation showed excellent stability: mean CV% across metrics was 1.95 ​% (Fig. S12, Table S8). Precision, the optimisation target, had a mean of 0.904 (CV% ​= ​1.876 ​%, 95 ​% CI [0.892, 0.916]), indicating robustness against overfitting from augmentation.

The optimal hyperparameters trained the final model on the entire internal training set (n ​= ​1388). Performance was evaluated sequentially on the internal test set (n ​= ​348) and then on the independent, un-augmented validation set (n ​= ​218).

#### 2.7.4 Feature importance and biological interpretation

To assess the biological relevance of ARTs, Pearson correlation analysis was conducted between ART and TRT features (significance at p ​< ​0.05). The relative contribution of features to drought tolerance prediction was evaluated using SHAP (SHapley Additive exPlanations) analysis on the Random Forest model trained with the complete feature set. SHAP values, measuring each feature's contribution while accounting for interactions [56], were averaged to determine overall importance (Fig. S16). Based on these results and the algorithmic logic of ART extraction, a conceptual model was developed linking key ARTs to established drought adaptation mechanisms (Fig. S15). This framework helps translate algorithmic outputs into biologically meaningful root architectural strategies. Trait stability was assessed by calculating coefficients of variation for ART and TRT features across experimental conditions (Figs. S9 and S13).

## 3 Results

### 3.1 Drought Tolerance Characterisation

#### 3.1.1 Genotype ranking and validation

**Fig. 2:**
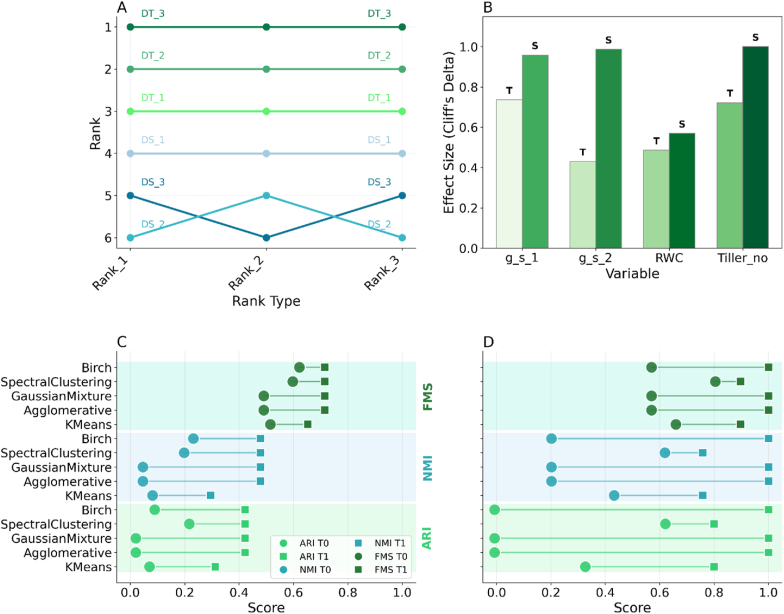
Drought tolerance characterisation and validation of wheat genotypes. (A) A bump chart illustrates the ranking of drought-tolerant (DT, green hues) and drought-susceptible (DS, blue hues) genotypes based on three physiological ranking methods. DT genotypes consistently rank higher (1–3) than DS genotypes. (B) Bar chart showing the effect size (Cliff's Delta) of drought on key physiological traits. The greater effect on susceptible (S) genotypes compared to tolerant (T) genotypes confirms their higher sensitivity to stress. (C, D) Dumbbell plots comparing external validation scores for five unsupervised clustering algorithms. In these plots, scores under control (T0) conditions are represented by circles, and scores under drought (T1) conditions are represented by squares, as defined in the legend of panel (C). Each line connects the T0 (circle) and T1 (square) scores for a specific algorithm on a given metric (Adjusted Rand Index [ARI], Normalised Mutual Information [NMI], and Fowlkes-Mallows Score [FMS]). The general rightward shift from T0 to T1 indicates that the algorithms achieved better separation of tolerant and susceptible genotypes under drought stress. (D) The separation between tolerant and susceptible groups is further enhanced under drought (T1) after fine-tuning the validation to account for the specific response of genotype DT_3.

The genotype ranking based on physiological responses to drought (RANK_1, RANK_2, RANK_3) consistently ranked genotypes (DT_1, DT_2, DT_3) as drought tolerant (i.e., higher ranks) than susceptible genotypes (DS_1, DS_2, DS_3). Minor rank swaps occurred between DS_2 and DS_3, but both consistently ranked as drought-susceptible, confirming their established classifications in the literature [[57], [58], [59], [60]] (Fig. 2A).

**Fig. 3:**
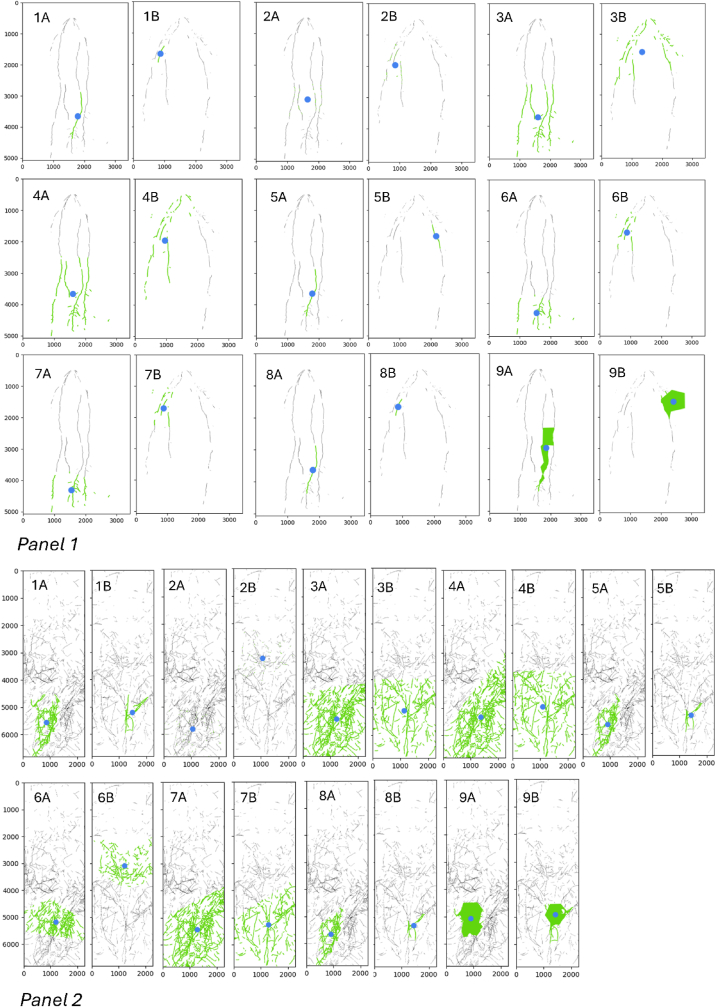
Visualisation of Algorithmic Root Traits (ART). Panel 1 displays results from glasshouse rhizotron imagery, while **Panel 2** exhibits field minirhizotron imagery. The figures highlight the largest dense root clusters (_density_points) in green, with the blue dots indicating the cluster centroids (_centre_x and _centre_y). Nine algorithms, detailed in Table 1, are represented by numbered overlays, illustrating how each algorithm identifies and locates the largest dense root cluster. The algorithms include: 1. DBSCAN, 2. Custom, 3. FCM, 4. GMM, 5. HDBSCAN, 6. K-means, 7. Mean-shift, 8. OPTICS, and 9. SLIC. In each panel, (A) corresponds to the drought-tolerant genotype DT_1, and (B) corresponds to the drought-susceptible genotype DS_3. For example, Panel 1 (6A) shows the K-means algorithm applied to the drought-tolerant genotype DT_1, and Panel 1 (6B) shows the same algorithm applied to the drought-susceptible genotype DS_3. The green areas represent the largest dense root clusters, and the blue dots indicate their centers as identified by the respective algorithms.

#### 3.1.2 Physiological responses to drought

Significant differences (ANOVA/Kruskal-Wallis, p ​< ​0.0001) were observed in stomatal conductance (g_s_1, g_s_2), relative water content (RWC), and tiller number (Tiller_no) between drought-tolerant and susceptible genotypes under stress. Cliff's Delta effect sizes indicated larger negative impacts of drought on the susceptible group for all measured physiological traits, highlighting these traits as robust indicators of drought response (Fig. 2B).

#### 3.1.3 Clustering genotype for drought tolerance

Unsupervised clustering of genotypes based on physiological traits yielded moderate separation between susceptible and tolerant groups under control conditions (T0), with mean internal validation metrics such as Silhouette Score ∼0.45, Dunn Index ∼0.53, Calinski-Harabasz Index ∼21.8, and Davies-Bouldin Index ∼0.89 (see Table S6 for validation metrics). Cluster separation improved under drought conditions (T1), with mean Silhouette Score increasing to ∼0.50, Dunn Index to ∼1.00, Calinski-Harabasz Index to ∼30.4, and Davies-Bouldin Index decreasing to ∼0.65 (Table S6). External validation metrics (Adjusted Rand Index, Normalised Mutual Information, Fowlkes-Mallows Score) also showed improved cluster accuracy under T1 (Fig. 2C). This separation became more pronounced when accounting for the specific response of genotype DT_3, suggesting some intra-group variability within the drought-tolerant classification (Fig. 2D).

### 3.2 Algorithmic Root Trait (ART) extraction results

#### 3.2.1 ART extraction process and visualisation

The ART approach, utilising eight unsupervised ML algorithms and the Custom algorithm (Table 1), yielded 27 ARTs (Table S5). Each algorithm generated three ARTs from the largest identified dense root cluster in an image: [AlgorithmName]_density_points (cluster size in pixels), [AlgorithmName]_centre_x (horizontal centroid coordinate), and [AlgorithmName]_centre_y (vertical centroid coordinate). Fig. 3 visually demonstrates how each of the nine algorithms uniquely identifies these dense root clusters and their central locations within root images from both glasshouse (Panel 1, 41 DAS) and field (Panel 2, 147 DAS) experiments for genotypes DT_1 (tolerant) and DS_3 (susceptible).

### 3.3 Comparative analysis: ART vs. TRT

#### 3.3.1 Feature space analysis and multivariate comparisons

**Fig. 4:**
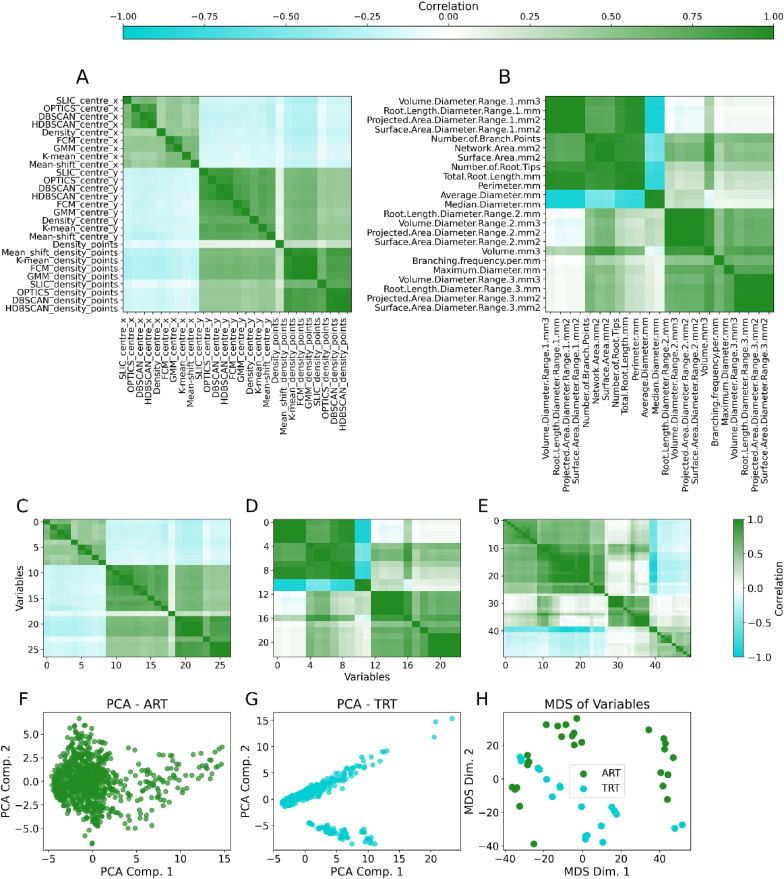
Comparison between ART and TRT datasets. (A) Correlation matrix heatmap of ARTs. (B) Correlation matrix heatmap of TRTs. (C) Euclidean distance matrix heatmap of ARTs. (D) Euclidean distance matrix heatmap of TRTs. (E) Euclidean distance matrix heatmap for the Combined dataset. (F) PCA plot of ARTs. (G) PCA plot of TRTs. (H) MDS plot comparing ARTs and TRTs.

The ART dataset exhibited higher internal variability, with a mean Euclidean distance among its features of 35.59 (SD: 11.41), compared to the TRT dataset's 28.91 (SD: 14.28). This suggests ARTs capture more complex and discriminative architectural patterns. Correlation Matrix Heatmaps (Fig. 4A and B) and Euclidean Distance Matrix Heatmaps (Fig. 4C and D) visually supported these findings, with 10.13039/100007725ART features generally displaying greater dissimilarity (darker colours in Euclidean distance heatmap). The combined dataset had a mean Euclidean distance of 34.81 (SD: 11.90) (Fig. 4E). Principal Component Analysis (PCA) plots showed ART variables distributed more broadly across components (Fig. 4F), unlike TRT variables where variance was more concentrated in the first component (Fig. 4G). Multidimensional Scaling (MDS) plots mirrored these structural differences (Fig. 4H). A Permutational Multivariate Analysis of Variance (PERMANOVA) confirmed a significant difference between the ART and TRT datasets (Test Statistic: 5.578, p-value: 0.002).

### 3.4 Drought tolerance classification performance

#### 3.4.1 Algorithm selection and performance comparison

**Fig. 5:**
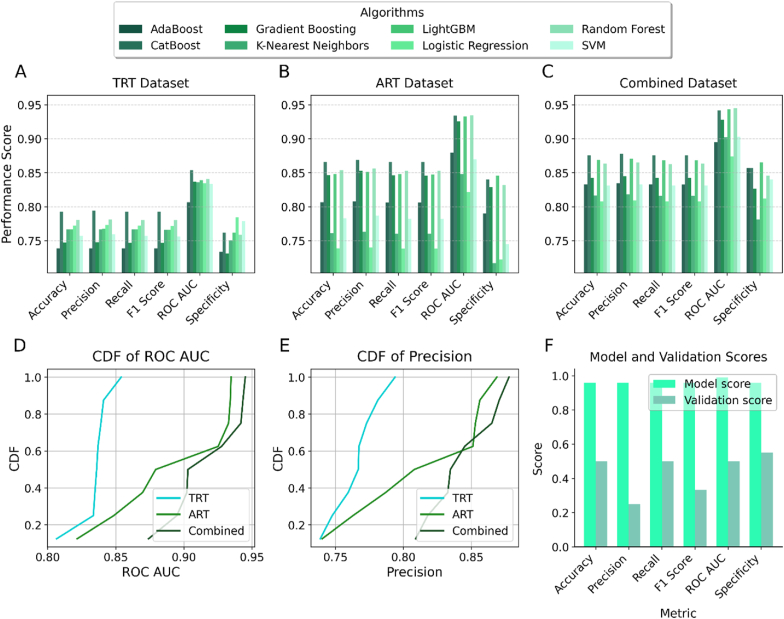
Comparative performance of classification algorithms and final model validation. (A–C) Grouped bar charts comparing the performance of eight classification algorithms across six key metrics on the (A) TRT, (B) ART, and (C) combined datasets. For each metric, Random Forest and CatBoost consistently rank among the top performers. (D–E) Cumulative Distribution Function (CDF) plots illustrating the distribution of (D) ROC AUC and (E) Precision scores, showing that models trained on the combined dataset consistently outperform those trained on ART or TRT alone. (F) Performance of the final selected Random Forest model, comparing its scores on the internal development data ("Model score") with its scores on the completely independent validation set ("Validation score"), demonstrating robust generalisation.

Among the eight classification algorithms tested, CatBoost and Random Forest generally exhibited superior performance across TRT, ART, and the combined datasets, as shown by the grouped bar chart comparisons (Fig. 5A–C). The cumulative distribution scores for ROC AUC and precision confirmed that models trained on the combined dataset consistently outperformed those built with either ART or TRT alone (Fig. 5D and E). Based on its strong and stable performance, Random Forest was selected for all subsequent detailed modeling and analysis. The final tuned Random Forest model's excellent performance on the internal development data ("Model score") and its robust generalisation to the independent validation set ("Validation score") are illustrated in Fig. 5F.

### 3.5 Model performance and validation

Performance was assessed on internal test sets derived from augmented development datasets.

**Fig. 6:**
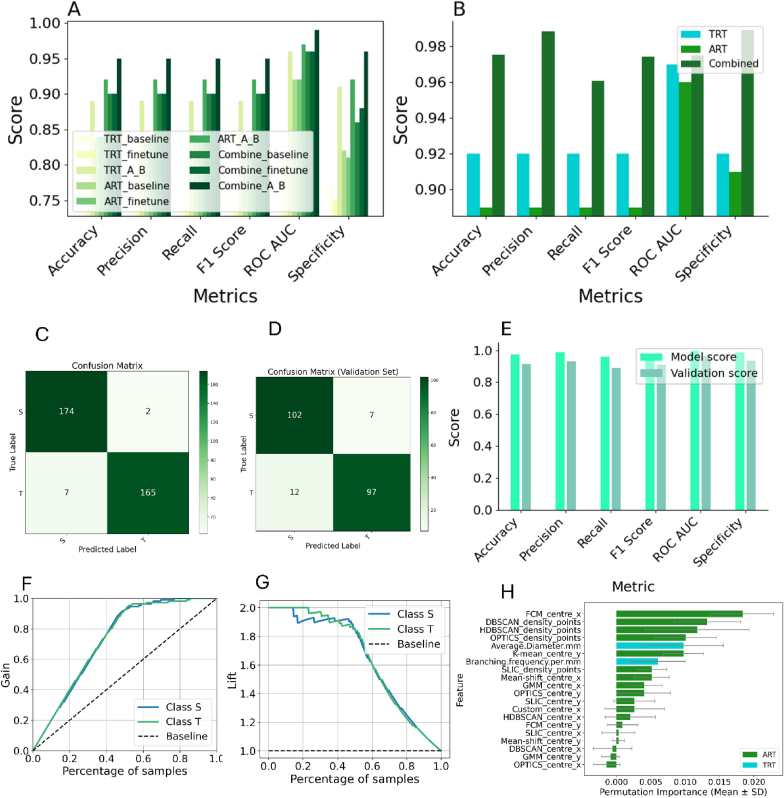
Performance of Random Forest classifiers in classifying drought tolerance using different datasets. (A) Grouped bar charts illustrating the step-wise improvement of the Random Forest model's performance. For each data type (TRT, ART, Combine), performance is shown for the baseline, fine-tuned, and augmented (A_B) model versions. (B) Bar chart comparison of the final, optimised models for the TRT, ART, and combined datasets, highlighting the superior performance of the model trained on the combined dataset. (C) Confusion matrix for the best-performing model on the internal test set. (D) Confusion matrix for the best-performing model on the independent validation set. (E) The best-performing model's metric scores during development ("Model score") and on the validation dataset ("Validation score"). (F) Cumulative gain curve for the validation dataset. (G) Lift curve for the validation dataset. (H) Permutation feature importance of the final model.

**Classification with TRTs:** The optimised Random Forest model trained on the augmented TRT dataset (TRT_A_B) achieved an accuracy of 0.856, precision of 0.860, and ROC AUC of 0.927 on its internal test set (Table S10). The step-wise improvement from baseline to the final augmented model is visually represented in the bar charts of Fig. 6A.

**Classification with ARTs:** The model trained on the augmented ART dataset (ART_A_B) yielded superior performance, with an accuracy of 0.963, precision of 0.963, and ROC AUC of 0.997 on its internal test set (Table S10, Fig. 6A).

**Classification with Combined Root Traits (TRT and ART):** The Random Forest model trained on the combined, augmented dataset (Combine_A_B) demonstrated the highest performance on its internal test set, with an accuracy of 0.974, precision of 0.975, and ROC AUC of 0.998 (Table S10). A direct comparison in Fig. 6B clearly illustrates that the model using the combined dataset outperforms the models trained on TRT or ART alone across most metrics. Confusion matrices for this model on the internal test set showed high true positive and true negative rates (Fig. 6C).

#### 3.5.1 Independent validation results

The optimal Random Forest model (trained on Combine_A_B) was evaluated on the completely independent, un-augmented validation set (n ​= ​218, 50 variables). It achieved a robust performance: accuracy 0.91, precision of 0.93, recall of 0.89, F1 score of 0.91, and ROC AUC of 0.96 (Fig. 6D and E). Cumulative gains and lift curves further confirmed its effectiveness in distinguishing tolerant and susceptible genotypes (Fig. 6F and G).

### 3.6 Biological relevance and feature contribution

#### 3.6.1 ART feature contribution and biological interpretation

##### 3.6.1.1 Contribution of ART in drought tolerance classification

SHAP analysis revealed that ART features accounted for 73.3 ​% of the combined model's predictive power compared to only 26.7 ​% for TRTs (Fig. S14), despite representing just 54 ​% of total features. Algorithm-level permutation importance confirmed ART dominance, with FCM (0.019), OPTICS (0.013), and HDBSCAN (0.012) showing the strongest individual contributions to model performance (Table S7).

##### 3.6.1.2 Biological interpretation and mechanism links to drought adaptation

**Fig. 7:**
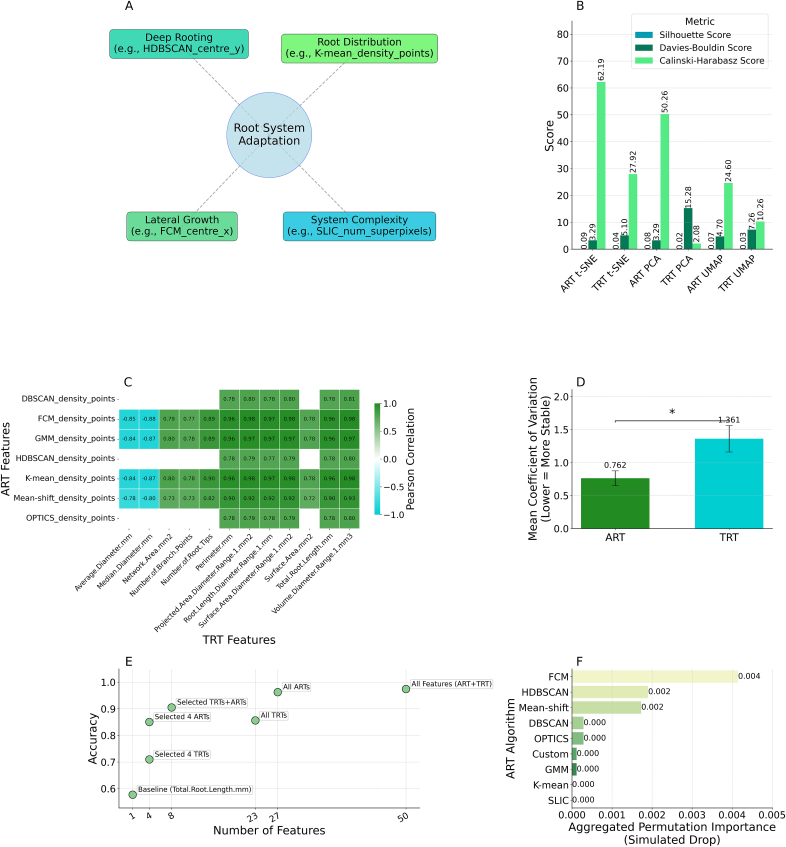
Biological relevance and performance advantages of ART. (A) Drought adaptation mechanisms captured by ART features, showing connections to established physiological processes; (B) Performance metrics comparison between ART and TRT across multiple evaluation criteria; (C) Significant correlations between ART features and established TRT metrics (|r| ​≥ ​0.6, p ​< ​0.05); (D) Trait stability comparison showing significantly lower coefficient of variation for ART versus TRT across experimental conditions (p ​= ​0.0137); (E) Model accuracy versus feature count, demonstrating the higher information density of ARTs compared to TRTs; (F) Algorithm ablation study results, showing the relative contributions of different algorithms to model performance, with FCM, HDBSCAN, and Mean-shift providing the greatest contributions.

Strong correlations between ART and TRT features (Fig. 7C, Fig. S6) established the biological validity of algorithmically-derived traits. Notably, 66 out of 129 significant correlations (51 ​%) exceeded |r| ​= ​0.7, demonstrating robust connections between computational and traditional measurements. Root mass clustering ARTs (FCM_density_points, GMM_density_points, K-mean_density_points) showed exceptional correlations with TRT volume metrics (r ​= ​0.93–0.98, p ​< ​0.05), validating _density_points as precise quantifiers of localised root biomass. Vertical position traits (_centre_y) correlated positively with depth metrics (r ​= ​0.60–0.68, p ​< ​0.05), while density clustering traits (_density_points) showed significant negative correlations with diameter metrics (r ​= ​−0.85 to −0.88, p ​< ​0.05), indicating adaptive trade-offs between root biomass concentration and construction costs.

Feature importance analysis identified FCM_centre_x (SHAP: 0.071) and HDBSCAN_density_points (SHAP: 0.071) as the most influential predictors, with HDBSCAN_centre_y (SHAP: 0.024) also ranking highly (Fig. S16, Table S9). Complementary analysis using feature-level permutation importance further highlighted the significance of features such as FCM_centre_x, DBSCAN_density_points, and HDBSCAN_density_points (Fig. 6H). These findings establish a clear mechanistic pathway: ARTs quantify specific architectural adaptations—deeper rooting (_centre_y), enhanced lateral exploration (_centre_x), and strategic biomass allocation (_density_points)—that enable efficient water acquisition during drought stress. This enhanced water uptake maintains favorable plant water status, supporting higher stomatal conductance and ultimately conferring superior drought tolerance. This causal framework explains why ART-based models excel at classifying genotypes initially grouped by physiological drought responses and aligns with established root ideotypes for water-limited environments [43] (Fig. 7A).

#### 3.6.2 Model performance and stability assessment

##### 3.6.2.1 Trait stability assessment

ART features demonstrated significantly superior stability across experimental conditions (glasshouse, field, all) compared to TRTs, with a lower mean Coefficient of Variation (CV) (ART mean CV: 0.762 vs. TRT mean CV: 1.361, Welch's *t*-test p ​= ​0.0137) (Fig. 7D, Figs. S9 and S13).

##### 3.6.2.2 Model performance advantages and information density of ARTs

The comparative analysis demonstrates that ARTs significantly outperform TRTs for drought tolerance classification (Fig. 7B). Models using all 27 ARTs achieved 96.3 ​% accuracy and 99.7 ​% ROC AUC, substantially better than models using all 23 TRTs (85.6 ​% accuracy, 92.7 ​% ROC AUC)—a 10.7 percentage point improvement. Even just 4 selected ARTs matched the performance of all 23 TRTs, representing 5.8 ​× ​higher information density (0.213 vs. 0.037 accuracy/feature) (Fig. 7E–Table S10). This efficiency has practical implications for high-throughput phenotyping, where extracting fewer, more informative traits can significantly accelerate screening. The superior performance of ARTs stems from their ability to capture spatial patterns of root organisation that global TRTs miss. ARTs also demonstrated significantly higher stability across environments (mean CV ​= ​0.762 vs. 1.361 for TRTs, p ​= ​0.0137), enhancing their reliability for field applications. When combined, ARTs and TRTs showed synergistic effects, with just 8 features (4 ARTs ​+ ​4 TRTs) achieving 89.9 ​% accuracy, further confirming the complementary nature of these trait types and their collective value for drought tolerance screening (Table S10).

##### 3.6.2.3 Cross-validation stability results

The Random Forest model selected via 10-fold cross-validation on the augmented training data demonstrated excellent stability during the model selection process. The cross-validated precision (our optimisation target) averaged 0.904 (95 ​% CI: [0.892, 0.916]) with minimal variation across folds (CV% ​= ​1.876 ​%). This stability was consistent across all key performance metrics, with coefficients of variation ranging from 1.242 ​% (ROC AUC) to 2.591 ​% (F1 Score), as detailed in Fig. S12 and Table S8. The final model, trained on the augmented training data, achieved excellent performance on the internal test set (accuracy: 0.974, precision: 0.975, F1 score: 0.973, ROC AUC: 0.998) and, importantly, when tested on a completely independent, un-augmented validation set, it achieved an accuracy of 0.913, precision: 0.933, F1 score: 0.911, and ROC AUC: 0.962, confirming its robustness and generalisability.

#### 3.6.3 Comparative validation results

##### 3.6.3.1 Baseline comparisons

To further contextualise the performance of ARTs and TRTs, a simpler baseline was established by training a Random Forest model using only the total pixel count from each segmented root mask as a single feature. This “Total Root Pixels” model, representing the overall scale of the root system, achieved an accuracy of 57.8 ​% and a ROC AUC of 61.7 ​% (Table S10). The substantially higher performance of models incorporating multiple TRTs (e.g., “All TRTs” model: 85.6 ​% accuracy) and particularly ARTs (e.g., “All ARTs” model: 96.3 ​% accuracy; “Selected 4 ARTs” model: 85.1 ​% accuracy) demonstrates that these more sophisticated trait sets capture critical architectural and distributional information for drought tolerance classification that extends significantly beyond simple root system size.

##### 3.6.3.2 t-SNE comparison results and algorithm contribution

The superior ability of ART features to distinguish between drought-tolerant and susceptible genotypes was visually evident in t-SNE projections (Fig. S17), which showed significantly better group separation compared to TRT features. Specifically, ART features showed a 109 ​% greater Silhouette Score (0.092 vs. 0.044), 34 ​% better Davies-Bouldin Score (3.301 vs. 5.004, lower is better), and 113 ​% greater Calinski-Harabasz Score (61.512 vs. 28.874) compared to TRT features. This pattern of improved separation was consistent across all dimensionality reduction techniques tested (t-SNE and PCA), confirming that ARTs capture distinct information relevant to drought tolerance classification.

The different cluster distributions between ART and TRT feature spaces suggest these approaches capture complementary information about root architecture, explaining why their combination yields superior classification performance (97.4 ​% accuracy) compared to either feature set alone. Algorithm ablation study results show the relative contributions of different algorithms to model performance, with FCM, HDBSCAN, and Mean-shift providing the greatest contributions (Fig. 7F).

## 4 Discussion

This study introduces the ART extraction method, a novel computational framework that significantly advances the capacity to extract nuanced, physiologically relevant information from plant root images. By employing an ensemble of unsupervised ML algorithms and the Custom algorithm applied to segmented binary images, ART uncovers spatially-explicit architectural patterns, particularly the characteristics of dense root clusters. These traits, "latent" in the sense that their specific attributes (e.g., precise location, boundaries, density) emerge from algorithmic analysis rather than being predefined by direct visual rules, proved remarkably effective.

ART-based models substantially outperformed traditional approaches (96.3 ​% vs. 85.6 ​% accuracy) while achieving 5.8 ​× ​higher information density, demonstrating the significant value of algorithmically-derived traits for drought tolerance classification. This enhanced efficiency and discriminative power has profound implications for high-throughput phenotyping, offering a pathway to accelerate genetic screening for complex stress resilience traits.

The superior predictive power of ARTs stems from their ability to quantify biologically meaningful root system adaptations to water-limited environments, phenomena well-documented in plant physiology [43,45,61,62]. The analyses suggest a clear mechanistic link between these algorithmically-derived traits and drought resilience, as captured by SHAP analysis where ARTs accounted for 73.3 ​% of the combined model's predictive power (Fig. S14). For instance, vertical distribution traits like HDBSCAN_centre_y (SHAP importance ∼0.024), which correlated significantly with TRT depth metrics (r ​= ​0.60–0.68, p ​< ​0.05), directly quantify deeper rooting—a key component of Lynch's (2013) "Steep, Cheap, and Deep" ideotype. Similarly, lateral exploration traits such as FCM_centre_x (the most influential single feature, SHAP ∼0.071), which showed strong negative correlations with TRT average diameter metrics (r ​= ​−0.85 to −0.88, p ​< ​0.05), likely represent the adaptive trade-off between extensive soil foraging and root construction costs under resource scarcity [63]. Furthermore, _density_points traits, exemplified by HDBSCAN_density_points (second most important feature, SHAP ∼0.071), quantify strategic root biomass clustering. These correlated strongly with TRT volume/mass metrics (r ​= ​0.90–0.98, p ​< ​0.05) and reflect adaptive resource allocation where genotypes concentrate root development in resource-rich zones, indicative of root plasticity [35]. This capacity of ARTs to characterise the precise location and intensity of dense root congregations—putative primary water uptake zones [64]—at a different spatial resolution and perspective than global TRTs, likely explains their dominant predictive contribution. The analyses suggest a plausible biological interpretation: (i) ARTs quantify specific architectural adaptations (deep rooting via _centre_y, lateral exploration via _centre_x, strategic biomass allocation via _density_points); (ii) these adaptations are associated with more efficient water acquisition during drought; (iii) this enhanced water acquisition maintains favorable plant water status; (iv) improved water status supports higher stomatal conductance and leaf relative water content; and (v) these physiological responses confer greater drought tolerance, thereby explaining the superior performance of ART-based models in classifying genotypes initially grouped by these physiological indicators.

Despite the predictive dominance of ARTs, TRTs remain valuable, exhibiting a synergistic relationship when combined. The integrated ART ​+ ​TRT model achieved the highest classification accuracy (97.4 ​%), surpassing models based exclusively on ARTs (96.3 ​%) or TRTs (85.6 ​%) (Fig. 6B–Table S10). This synergy is further evidenced by the distinct data structures revealed by multivariate analyses (PCA and MDS in Fig. 4F–H; t-SNE in Fig. S17) and PERMANOVA (Test Statistic: 5.578, p ​= ​0.002), indicating that ARTs and TRTs capture different, complementary facets of root system architecture. The t-SNE visualisations showed ART features provided substantially better group separation than TRTs (Fig. S17). Even a streamlined model combining just four selected ARTs with four TRTs achieved comparable performance, demonstrating practical utility for efficient phenotyping.

Beyond its enhanced predictive accuracy, the ART methodology presents significant technical advantages critical for robust scientific inquiry and practical deployment. Notably, ART features demonstrated superior stability across diverse experimental settings (mean CV 0.762 vs 1.361 for TRTs, p ​= ​0.0137; Fig. 7D, Fig. S9), a crucial attribute for reliable phenotyping in variable field conditions. The ensemble nature of ART, employing nine distinct algorithms with diverse mathematical principles (Table 1), ensures comprehensive trait capture while yielding interpretable intermediate traits (e.g., FCM_centre_x, HDBSCAN_density_points). While ablation studies confirmed that a core trio of algorithms (FCM, HDBSCAN, and Mean-shift) provided ∼80 ​% of the predictive gain (Fig. 7F–Table S7), the full ensemble offers superior robustness, justifying this approach over single-algorithm reliance. This interpretability is pivotal for hypothesis-driven research, offering a clear advantage over "black-box" models by allowing direct investigation of specific root architectural components. Moreover, ART's modular design facilitates customisation and future development: researchers can use the core trio of algorithms for rapid screening, incrementally add others for deeper insights, or integrate new algorithms to target novel root characteristics, ensuring the framework's enduring relevance.

The ART framework signifies a conceptual advancement in image-based phenotyping, shifting beyond predefined geometric descriptors to algorithmically discover and quantify biologically meaningful features. Unlike methods focusing on global distribution comparisons (e.g., Peeples et al. [31]) abstract topology (e.g., Li et al. [32]), supervised landmark detection (e.g., Berrigan et al. [29]), or temporal embeddings (e.g., Ubbens et al. [30]), ART extracts localised, geometric and density traits from algorithmically-identified dense regions (Table S1). This emphasis on explainable features (Fig. 7A and C) distinguishes ART from "black-box" deep learning models where the basis of prediction can be opaque [65]. ART thus bridges traditional trait extraction and machine learning, offering a powerful yet understandable means to leverage unsupervised learning for phenotype discovery. Beyond root phenotyping, this framework could be adapted to quantify plant disease symptoms or other complex visual traits with enhanced objectivity.

While this initial study focused on method development using six wheat genotypes, robustly demonstrating ART's discriminative power based on established drought responses (Fig. 2A and B) [[57], [58], [59], [60]], future work should validate ART across diverse genotypes, species (e.g., maize, rice), and environmental conditions to establish its broader applicability.

The ART framework is inherently extensible, with all code and parameters available on GitHub for replication and adaptation. Future development could involve applying algorithms directly to RGB images to leverage colour and intensity information [[66], [67], [68]], incorporating texture analysis or advanced shape descriptors [69,70], adapting ARTs for regression modeling, or refining parameter optimisation through Monte Carlo simulations [71]. These extensions could potentially reduce dependency on initial segmentation while expanding analytical capabilities.

Crucially, the substantial predictive power of ARTs (73.3 ​% contribution in combined models, Fig. S14) offers exciting prospects for multi-omics integration. By quantitatively linking these nuanced root phenotypes to genomic, transcriptomic, or metabolomic data, ARTs could facilitate the identification of genetic markers and pathways underpinning drought tolerance and other complex traits.

In conclusion, the ART method provides a novel, objective, scalable, and customisable toolset that augments traditional root phenotyping pipelines. By enabling researchers to delve deeper into the complexities of plant adaptation, this work provides a conceptual blueprint for harnessing latent information within image data. It demonstrates that by relying on human vision alone, we may not truly see what we perceive [6]. By empowering us to 'see the unseen' through algorithmic lenses, this approach prompts a fundamental question for biology: what other critical patterns lie hidden, just beyond the limits of our perception?

## Author contributions

MS designed and conducted the experiment. SK guided in refining the experiments. MS wrote the manuscript and developed the code. CT and MH contributed to the conceptual ideas of the manuscript. AD created Fig. S2 and provided guidance in optimising image capture. All reviewed and edited the manuscript.

## Funding

This study was funded by 10.13039/501100018823Agriculture Victoria Research, Victoria state government, Australia.

## Data availability

All code, data and segmented images are available for download at https://github.com/shoaibms/ART.

## Declaration of competing interest

The authors declare that they have no known competing financial interests or personal relationships that could have appeared to influence the work reported in this paper.

## References

[1] Seethepalli A., Dhakal K., Griffiths M., Guo H., Freschet G.T., York L.M. (2021). RhizoVision explorer: open-source software for root image analysis and measurement standardization. AoB PLANTS.

[2] Tajima R., Kato Y. (2011). Comparison of threshold algorithms for automatic image processing of rice roots using freeware ImageJ. Field Crops Res..

[3] WinRHIZO (2024). https://regent.qc.ca/assets/winrhizo_software.html.

[4] Yasrab R., Atkinson J.A., Wells D.M., French A.P., Pridmore T.P., Pound M.P. (Nov 1 2019). RootNav 2.0: deep learning for automatic navigation of complex plant root architectures. GigaScience.

[5] Das A. (2015). Digital imaging of root traits (DIRT): a high-throughput computing and collaboration platform for field-based root phenomics. Plant Methods.

[6] Hoffman D.D., Singh M., Prakash C. (2015). The interface theory of perception. Psychon. Bull. Rev..

[7] Misra I., Lawrence Zitnick C., Mitchell M., Girshick R. (2016). Presented at the Proceedings of the IEEE Conference on Computer Vision and Pattern Recognition, Las Vegas, NV, USA.

[8] Huang Z., Zeng Z., Liu B., Fu D., Fu J. (2020). Pixel-bert: aligning image pixels with text by deep multi-modal transformers. arXiv pre-print server.

[9] Schölkopf B. (2021). Toward causal representation learning. Proc. IEEE.

[10] Kirby K.N., Kosslyn S.M. (1990). Thinking visually. Mind Lang..

[11] White P.J., George T.S., Gregory P.J., Bengough A.G., Hallett P.D., McKenzie B.M. (Jul 2013). Matching roots to their environment. Ann. Bot..

[12] Jacobsen A.G., Jervis G., Xu J., Topping J.F., Lindsey K. (Jul 2021). Root growth responses to mechanical impedance are regulated by a network of ROS, ethylene and auxin signalling in Arabidopsis. New Phytol..

[13] Uga Y. (Feb 2021). Challenges to design-oriented breeding of root system architecture adapted to climate change. Breed. Sci..

[14] Lynch J.P. (1995). Root architecture and plant productivity. Plant Physiol..

[15] Tumber-Dávila S.J., Schenk H.J., Du E., Jackson R.B. (Aug 2022). Plant sizes and shapes above and belowground and their interactions with climate. New Phytol..

[16] Ober E.S. (2021). Wheat root systems as a breeding target for climate resilience. Theor. Appl. Genet..

[17] Yu P. (Apr 2021). Plant flavones enrich rhizosphere oxalobacteraceae to improve maize performance under nitrogen deprivation. Nat. Plants.

[18] Ruiz-Lozano J.M. (2016). Arbuscular mycorrhizal symbiosis induces strigolactone biosynthesis under drought and improves drought tolerance in lettuce and tomato. Plant Cell Environ..

[19] Lynch J.P., Chimungu J.G., Brown K.M. (2014). Root anatomical phenes associated with water acquisition from drying soil: targets for crop improvement. J. Exp. Bot..

[20] Maurel C., Nacry P. (2020). Root architecture and hydraulics converge for acclimation to changing water availability. Nat. Plants.

[21] Lynch J.P. (2022). Harnessing root architecture to address global challenges. Plant J..

[22] Rolfe S.A., Griffiths J., Ton J. (Jun 2019). Crying out for help with root exudates: adaptive mechanisms by which stressed plants assemble health-promoting soil microbiomes. Curr. Opin. Microbiol..

[23] Prince S.J. (2017). Root xylem plasticity to improve water use and yield in water-stressed soybean. J. Exp. Bot..

[24] Trachsel S., Kaeppler S.M., Brown K.M., Lynch J.P. (2011). Shovelomics: high throughput phenotyping of maize (zea mays L.) root architecture in the field. Plant Soil.

[25] Wasson A., Bischof L., Zwart A., Watt M. (Feb 2016). A portable fluorescence spectroscopy imaging system for automated root phenotyping in soil cores in the field. J. Exp. Bot..

[26] Huck M.G., Taylor H.M. (1982). The rhizotron as a tool for root research. Adv. Agron..

[27] Bauer F.M. (2022). Development and validation of a deep learning based automated minirhizotron image analysis pipeline. Plant Phenomics.

[28] Palta J.A., Turner N.C. (2019). Crop root system traits cannot be seen as a silver bullet delivering drought resistance. Plant Soil.

[29] Berrigan E.M. (2024). Fast and efficient root phenotyping via pose estimation. Plant Phenomics.

[30] Ubbens J., Cieslak M., Prusinkiewicz P., Parkin I., Ebersbach J., Stavness I. (2020). Latent space phenotyping: automatic image-based phenotyping for treatment studies. Plant Phenomics.

[31] Peeples J., Xu W., Gloaguen R., Rowland D., Zare A., Brym Z. (Jan 5 2023). Spatial and texture analysis of root system distribution with Earth mover's distance (STARSEED). Plant Methods.

[32] Li M., Frank M.H., Coneva V., Mio W., Chitwood D.H., Topp C.N. (Aug 2018). The persistent homology mathematical framework provides enhanced genotype-to-phenotype associations for plant morphology. Plant Physiol..

[33] Erenstein O., Jaleta M., Mottaleb K.A., Sonder K., Donovan J., Braun H.-J., Reynolds M.P., Braun H.-J. (2022). Wheat Improvement: Food Security in a Changing Climate.

[34] Gupta A., Rico-Medina A., Cano-Delgado A.I. (Apr 17 2020). The physiology of plant responses to drought. Science.

[35] Zhan A., Schneider H., Lynch J.P. (Aug 2015). Reduced lateral root branching density improves drought tolerance in maize. Plant Physiol..

[36] Odone A., Popovic O., Thorup-Kristensen K. (2023). Deep roots: implications for nitrogen uptake and drought tolerance among winter wheat cultivars. Plant Soil.

[37] Bacher H., Sharaby Y., Walia H., Peleg Z. (Mar 2 2021). Modifying root-to-shoot ratio improves root water influxes in wheat under drought stress. J. Exp. Bot..

[38] Bontpart T. (Sep 2020). Affordable and robust phenotyping framework to analyse root system architecture of soil-grown plants. Plant J..

[39] Duan S., Li B., Gu H., Jiang H., Zhang X., Liu X. (2022). Root matters: lying seeds flat with the crease Down improves grain yield in winter wheat under drought stress. Plant Soil.

[40] Python Software Foundation (2023). https://docs.python.org/3.12/reference/.

[41] R: A Language and Environment for Statistical Computing (2023). https://www.R-project.org/.

[42] Smith A.G. (Oct 2022). RootPainter: deep learning segmentation of biological images with corrective annotation. New Phytol..

[43] Lynch J.P. (2013). Steep, cheap and deep: an ideotype to optimize water and N acquisition by maize root systems. Ann. Bot..

[44] Lootens P., Ruttink T., Rohde A., Combes D., Barre P., Roldán-Ruiz I. (2016). High-throughput phenotyping of lateral expansion and regrowth of spaced Lolium perenne plants using on-field image analysis. Plant Methods.

[45] Hodge A. (2004). The plastic plant: root responses to heterogeneous supplies of nutrients. New Phytol..

[46] Wasson A.P. (2012). Traits and selection strategies to improve root systems and water uptake in water-limited wheat crops. J. Exp. Bot..

[47] Pedregosa F. (2011). Scikit-learn: machine learning in python. J. Mach. Learn. Res..

[48] Ester M., Kriegel H.-P., Sander J., Xu X. (1996). Presented at the Proceedings of the Second International Conference on Knowledge Discovery and Data Mining.

[49] Comaniciu D., Meer P. (2002). Mean shift: a robust approach toward feature space analysis. IEEE Trans. Pattern Anal. Mach. Intell..

[50] Ankerst M., Breunig M.M., Kriegel H.-P., Sander J. (1999). OPTICS: ordering points to identify the clustering structure. ACM Sigmod record.

[51] McInnes L., Healy J., Astels S. (2024, 2017). Hdbscan: hierarchical density based clustering. J. Open Source Softw..

[52] Bezdek J.C., Ehrlich R., Full W. (1984). FCM: the fuzzy c-means clustering algorithm. Comput. Geosci..

[53] Achanta R., Shaji A., Smith K., Lucchi A., Fua P., Süsstrunk S. (2012). SLIC superpixels compared to state-of-the-art superpixel methods. IEEE Trans. Pattern Anal. Mach. Intell..

[54] Smart R.E., Bingham G.E. (Feb 1974). Rapid estimates of relative water content. Plant Physiol..

[55] Raman A. (Dec 2012). Drought yield index to select high yielding rice lines under different drought stress severities. Rice.

[56] Lundberg S.M., Lee S.-I. (2017). A unified approach to interpreting model predictions. Adv. Neural Inf. Process. Syst..

[57] Bennani S., Nsarellah N., Jlibene M., Tadesse W. (2017). Efficiency of drought tolerance indices under different stress severities for bread wheat selection. Aust. J. Crop. Sci..

[58] Yadav A.K., Carroll A.J., Estavillo G.M., Rebetzke G.J., Pogson B.J. (Sep 24 2019). Wheat drought tolerance in the field is predicted by amino acid responses to glasshouse-imposed drought. J. Exp. Bot..

[59] Hone H. (2021/06/07 2021). Profiling, isolation and characterisation of beneficial microbes from the seed microbiomes of drought tolerant wheat. Sci. Rep..

[60] McDonald G. (2023). https://grdc.com.au/resources-and-publications/grdc-update-papers/tab-content/grdc-update-papers/2016/02/drought-tolerance-fo-wheat-varieties.

[61] Li X. (Sep 24 2019). Deeper roots associated with cooler canopies, higher normalized difference vegetation index, and greater yield in three wheat populations grown on stored soil water. J. Exp. Bot..

[62] Uga Y. (Sep 2013). Control of root system architecture by DEEPER ROOTING 1 increases rice yield under drought conditions. Nat. Genet..

[63] Comas L.H., Becker S.R., Cruz V.M., Byrne P.F., Dierig D.A. (Nov 5 2013). Root traits contributing to plant productivity under drought. Front. Plant Sci..

[64] Vadez V. (2014). Root hydraulics: the forgotten side of roots in drought adaptation. Field Crops Res..

[65] Castelvecchi D. (Oct 6 2016). Can we open the black box of AI?. Nat. News.

[66] Richardson A.D. (Jun 2019). Tracking seasonal rhythms of plants in diverse ecosystems with digital camera imagery. New Phytol..

[67] Johnson J. (2021). Enhanced field-based detection of potato blight in complex backgrounds using deep learning. Plant Phenomics.

[68] Pham N.-A. (Feb 27 2007). Quantitative image analysis of immunohistochemical stains using a CMYK color model. Diagn. Pathol..

[69] Kulkarni P., Karwande A., Kolhe T., Kamble S., Joshi A., Wyawahare M. (2021). Plant disease detection using image processing and machine learning. arXiv preprint.

[70] Das Choudhury S., Bashyam S., Qiu Y., Samal A., Awada T. (2018). Holistic and component plant phenotyping using temporal image sequence. Plant Methods.

[71] Shashaani S., Vahdat K. (2022). International Conference on Operations Research.

