# Supplementary material for: Seeing the unseen: A novel approach to extract latent plant root traits from digital images
Source: Plant Phenomics. 2025 Jul 9;7(3):100088. doi: 10.1016/j.plaphe.2025.100088 (PMC12709997; doi:10.1016/j.plaphe.2025.100088)
Supplement: Multimedia component 1 [file mmc1.docx]

SUPPLEMENTARY MATERIALS

Title

Seeing the Unseen: A Novel Approach to Extract Latent Plant Root Traits from Digital Images

**Authors**

Mirza Shoaib^1,2*,^ Adam M. Dimech^3^, Simone J Rochfort^2,3^, Christopher Topp^4^, Matthew J. Hayden^2,3^ and Surya Kant^5^*

**Affiliations**

^1^Agriculture Victoria, Grains Innovation Park, 110 Natimuk Road, Horsham, Victoria 3400, Australia

^2^School of Applied Systems Biology, La Trobe University, Bundoora, Victoria 3083, Australia

^3^Agriculture Victoria, AgriBio, Centre for AgriBioscience, 5 Ring Road, Bundoora, Victoria 3083, Australia

^4^Donald Danforth Plant Science Center, Saint Louis, Missouri, USA

^5^Department of Ecological, Plant and Animal Science, School of Agriculture, Biomedicine & Environment, La Trobe University, Bundoora, Victoria 3083, Australia

* Correspondence: [shoaib.mirza@agriculture.vic.gov.au](mailto:shoaib.mirza@agriculture.vic.gov.au) and [S.Kant@latrobe.edu.au](mailto:S.Kant@latrobe.edu.au)

**Table S1.** *Comprehensive comparison of ART with STARSEED, Persistent Homology, SLEAP, LSP, and TRTs. A detailed analysis of how ART differs from other prominent root phenotyping methods, comparing core computational philosophy/goals, primary algorithmic engines, input data types, nature of extracted traits/outputs, interpretability approach, custom algorithm/unique implementation details, primary applications demonstrated, and key differentiators from ART's perspective.*

| **Method/Framework Name (Reference)** | **Core Computational Philosophy/Goal** | **Primary Algorithmic Engine(s)** | **Input Data Type** | **Nature of Extracted 'Traits'/Outputs** | **Interpretability Approach** | **Custom Algorithm/Unique Implementation Detail** | **Primary Application Demonstrated** | **Key Differentiator from ART's Perspective** |
| --- | --- | --- | --- | --- | --- | --- | --- | --- |
| ART (this study) | Unsupervised discovery of significant root density patterns & their spatial locations | Ensemble of unsupervised clustering algos (K-means, DBSCAN, etc.) + Custom "Custom" | Segmented binary images | Geometric attributes of discovered dense root clusters (e.g., centre_y, density_points) | Linking cluster size/location to biological hypotheses (e.g., rooting depth, localised biomass) | "Custom" algorithm for densest cluster ID; Framework combining outputs from 9 diverse algorithmic perspectives | Root drought tolerance classification | Focuses on "what" & "where" dense root regions are via an exploratory, unsupervised ensemble; traits are interpretable spatial/density metrics. Complements & extends TRTs with localised data. |
| STARSEED (Peeples et al., 2023) | Comparing overall root system distributions via optimal transport | Earth Mover's Distance (EMD) | Pre-processed binary images | Scalar EMD score (global difference), Flow matrix (local changes between distributions) | Visualising flow matrix for local differences; CH index for group separation | Specific EMD cost function; Grid-based pre-feature extraction (e.g., root pixel %) for EMD input | Sesame root response to genotype/moisture | EMD provides a holistic distance metric between entire distributions; ART identifies specific dense clusters within a distribution. |
| Persistent Homology (Li et al., 2018) | Quantifying shape complexity via topological feature persistence | Persistent Homology (PH) | 2D point clouds (contours) | Persistence barcodes (H₀ for connected components, H₁ for 1D holes/loops), CV scores from barcode distances | Relating abstract topological features to architectural components; QTL mapping with PH-derived CV scores | Custom PH functions (e.g., Gaussian kernel density for leaf shape; distance function for root branch crossings) | Tomato leaf shape, serrations, root architecture | PH quantifies abstract topological features (connectivity, holes); ART quantifies geometric features (size, location) of dense pixel clusters. |
| SLEAP for Roots (Berrigan et al., 2024) | Supervised detection & grouping of pre-defined anatomical landmarks | Deep Learning (CNNs for pose estimation) | Raw RGB images | 2D coordinates of pre-defined root landmarks; Derived geometric traits (lengths, angles) | Direct biological meaning of landmarks & derived traits (e.g., primary root length, lateral root angle) | Adaptation of SLEAP (animal pose) to root landmarks; Specific root labelling protocols | Multi-species root topology & trait extraction from landmarks | SLEAP requires supervised labelling of known landmarks; ART is unsupervised, discovering regions of interest (dense clusters) without pre-defined landmark definitions. |
| Latent Space Phenotyping (Ubbens et al., 2020) | Learning discriminative low-dimensional embeddings of dynamic plant response to treatment | Variational Autoencoders (VAEs), CNNs, LSTMs | Raw RGB image sequences | Abstract n-dimensional embeddings; Geodesic path length in latent space (response-to-treatment magnitude) | Embeddings capture treatment effects; Path length quantifies response. Saliency maps for image region relevance. | CNN-LSTM encoder for classification, then VAE-like decoder to map latent space for path measurement | Plant response-to-treatment (drought, N-deficiency) over time | LSP learns an abstract latent space for overall plant response dynamics; ART extracts concrete, interpretable geometric traits from (typically) static images, focusing on root substructures. |
| Traditional Root Traits (TRTs) | Direct quantification of pre-defined, basic root morphological parameters | Image processing (e.g., thresholding, skeletonisation, pixel counting) | Segmented binary images | Basic geometric measures (e.g., total length, average diameter, area, convex hull, branching angles) | Direct measurement of visually obvious physical properties | Often relies on established algorithms in software like RhizoVision, ImageJ, WinRhizo | General root phenotyping | TRTs provide foundational, often global, metrics. ART aims to discover and quantify localised, potentially latent, density-based traits that complement and enhance TRTs for tasks like classification. |

**Table S2.** *List of genotypes used in the experiment.*

| **Drought Tolerance** | **Genotype Name** | **Abbreviation** | **Reference** |
| --- | --- | --- | --- |
| Tolerance | Gladius | DT_1 | Bennani et al. [57],[58] |
|  | DAS5_005489 | DT_2 | Hone et al. [59] |
|  | DAS5_CALINGIRI | DT_3 | Hone et al. [59] |
| Susceptible | Forrest | DS_1 | Glenn and Rebecca [60] |
|  | Hartog | DS_2 | Glenn and Rebecca [60] |
|  | DAS5_003811 | DS_3 | Hone et al. [59] |

**Table S3.** *Nutrient solution used in the glasshouse experiment.*

| **No** | **Nutrient** |  | | **Concentration** |
| --- | --- | --- | --- | --- |
| 1 | MgSO_4_ |  |  | 4 mM |
| 2 | CaCl_2_.2H_2_O |  |  | 4 mM |
| 3 | KH_2_PO_4_ pool |  |  | 3 mM |
|  | K_2_HPO_4_ pool |  |  |  |
| 4 | Fe^+^ (FeEDTANa Librel) |  |  | 400 µM |
| 5 | MnCl_2_.4H_2_O |  |  | 10 µM |
|  | ZnSO_4_.7H_2_O |  |  | 10 µM |
|  | CuSO_4_ |  |  | 2 µM |
|  | H_3_BO_3_ |  |  | 50 µM |
|  | Na_2_MoO_4_ |  |  | 0.2 µM |
| 6 | KNO_3_ |  |  | 1M |

**Table S4.** *Rhizovision settings for TRT extraction.*

| **Parameters** | **Settings** |
| --- | --- |
| RhizoVision Explorer Version | 2.0.3 |
| Root type | Broken roots |
| Image Thresholding Level | 255 |
| Invert images | false |
| Keep largest component | true |
| Filter noisy components on background | true |
| Maximum background noisy component size | 0.2 |
| Filter noisy components on foreground | false |
| Maximum foreground noisy component size | 1 |
| Enable edge smoothing | false |
| Edge smoothing threshold | 0 |
| Enable root pruning | true |
| Root pruning threshold | 5 |
| Convert pixels to physical units | true |
| Dots per inch | 600 |
| Pixel to millimeter conversion factor | 0.042333 |
| Diameter Range 1 | 0-0.3 |
| Diameter Range 2 | 0.3-0.6 |
| Diameter Range 3 | 0.6-above |
| Features output file name | features.csv |
| Save segmented images | true |
| Segmented image file name suffix |  |
| Save processed feature images | false |
| Processed image file name suffix |  |

**Table S5.** *List of ART and TRT variables.*

| **ART** | **TRT** |
| --- | --- |
| DBSCAN_density_points | Number.of.Root.Tips |
| DBSCAN_centre_x | Number.of.Branch.Points |
| DBSCAN_centre_y | Total.Root.Length.mm |
| Custom_density_points | Branching.frequency.per.mm |
| Custom_centre_x | Network.Area.mm2 |
| Custom_centre_y | Average.Diameter.mm |
| FCM_density_points | Median.Diameter.mm |
| FCM_centre_x | Maximum.Diameter.mm |
| FCM_centre_y | Perimeter.mm |
| GMM_density_points | Volume.mm3 |
| GMM_centre_x | Surface.Area.mm2 |
| GMM_centre_y | Root.Length.Diameter.Range.1.mm |
| HDBSCAN_density_points | Root.Length.Diameter.Range.2.mm |
| HDBSCAN_centre_x | Root.Length.Diameter.Range.3.mm |
| HDBSCAN_centre_y | Projected.Area.Diameter.Range.1.mm2 |
| K-mean_density_points | Projected.Area.Diameter.Range.2.mm2 |
| K-mean_centre_x | Projected.Area.Diameter.Range.3.mm2 |
| K-mean_centre_y | Surface.Area.Diameter.Range.1.mm2 |
| SLIC_density_points | Surface.Area.Diameter.Range.2.mm2 |
| SLIC_centre_x | Surface.Area.Diameter.Range.3.mm2 |
| SLIC_centre_y | Volume.Diameter.Range.1.mm3 |
| Mean-shift_density_points | Volume.Diameter.Range.2.mm3 |
| Mean-shift_centre_x | Volume.Diameter.Range.3.mm3 |
| Mean-shift_centre_y | |
| OPTICS_density_points | |
| OPTICS_centre_x |  |
| OPTICS_centre_y |  |

| 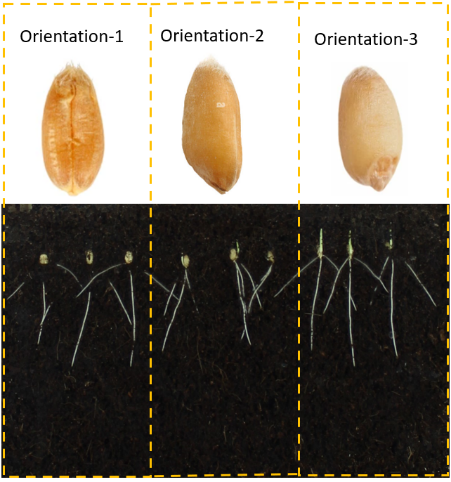 | 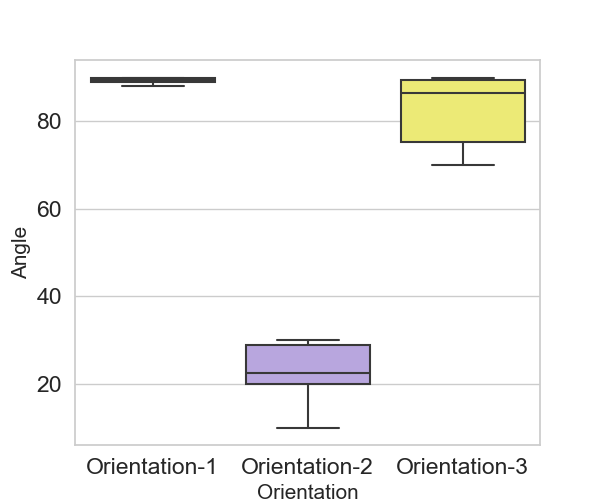 |
| --- | --- |
| A | B |

**Fig. S1.** *Effect of seed sowing orientation on root growth. (A) Different seed sowing orientations (Orientation-1, Orientation-2, Orientation-3) and the corresponding root growth patterns for each orientation. (B) Effect of seed sowing orientation on root angle. Orientation-1 = Brush pointed up and crease facing rhizotron wall, Orientation-2 = Brush pointed up and crease facing 90° away from rhizotron wall, Oriantation-3 = Brush pointed up and crease facing opposite (180°) to rhizotron wall.*

**
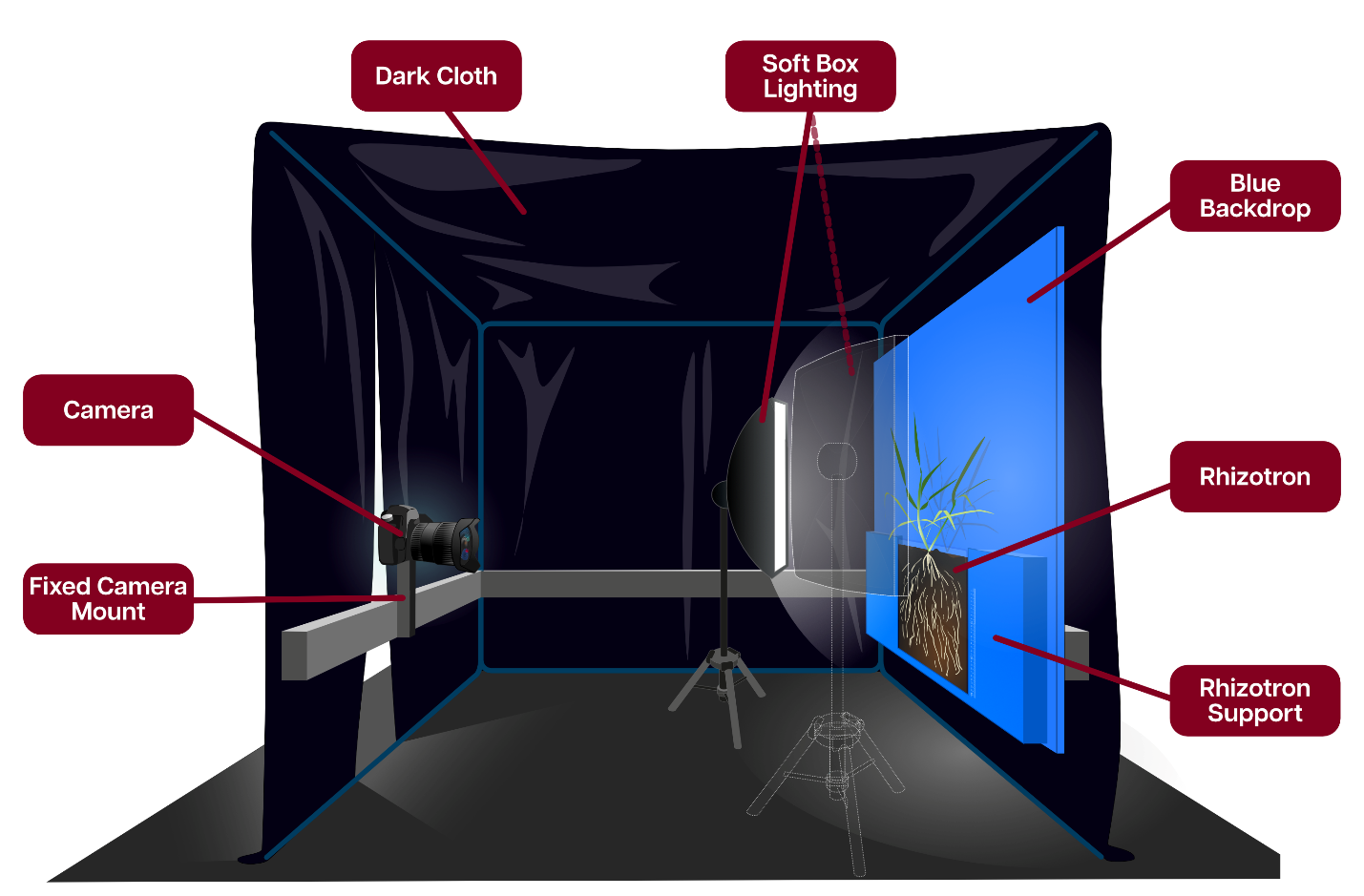
**

**Fig. S2.** *The imaging setup used for rhizotron experiments. A custom imaging set-up was built in the glasshouse to capture rhizotron images. To prevent reflectance from the rhizotron, a blackout curtain was used to form a dark tent, and a frame secured the rhizotron and camera at a consistent distance. The set-up was painted matt black, except for the light blue (HEX code: 0089b6) image background, and it was illuminated with four diffused studio lights angled to prevent reflection.*


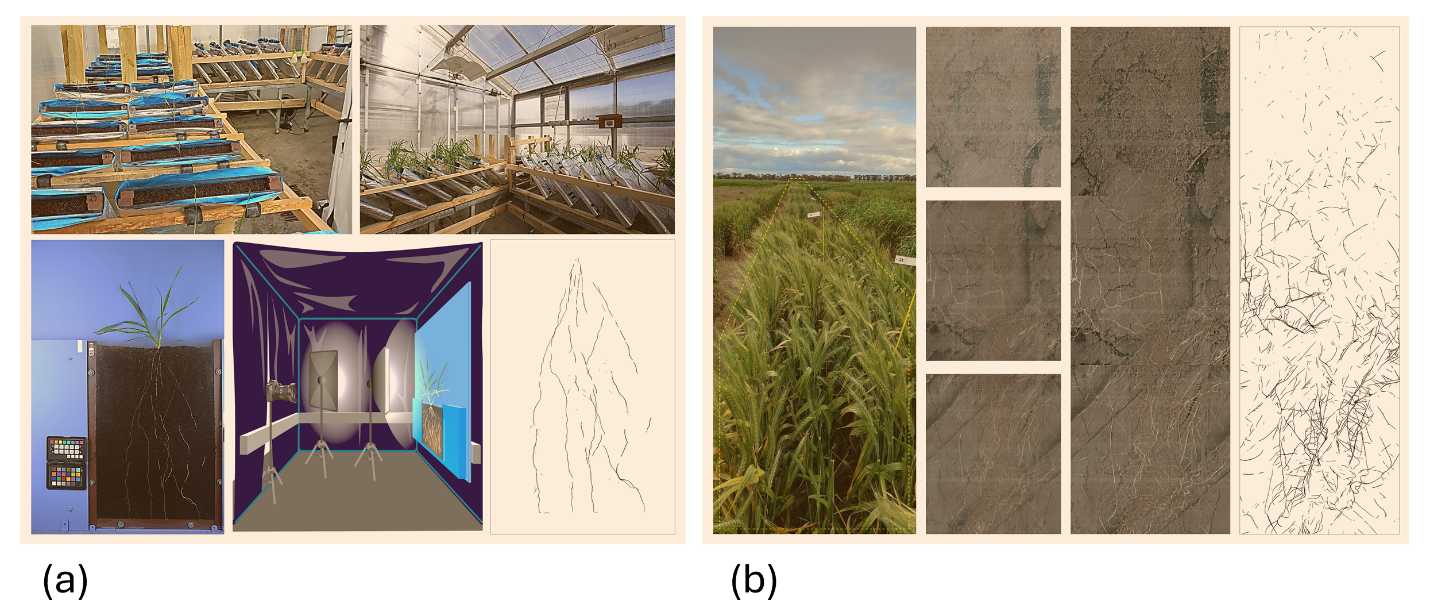


| A | B |
| --- | --- |

**Fig. S3.** *Experimental set-up. (A) Rhizotron experiment in glasshouse. The experimental set-up included a rhizotron holder, imaging station, rhizotron image, and image after cropping and segmentation. (B) Field experiment. Layout and view of the plots: Three sets of images captured from a single minirhizotron, three images after rotation and switching in the correct order, and a segmented image.*


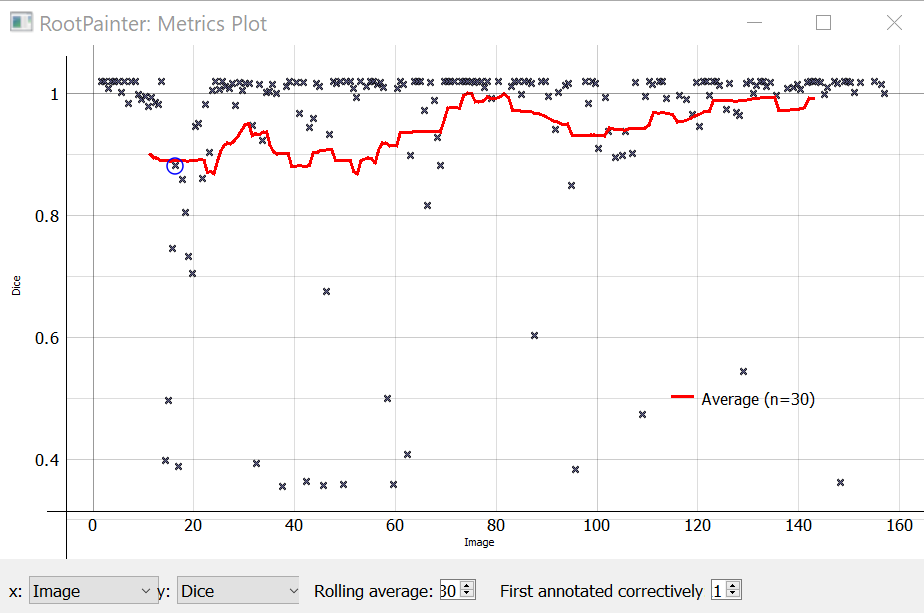


A


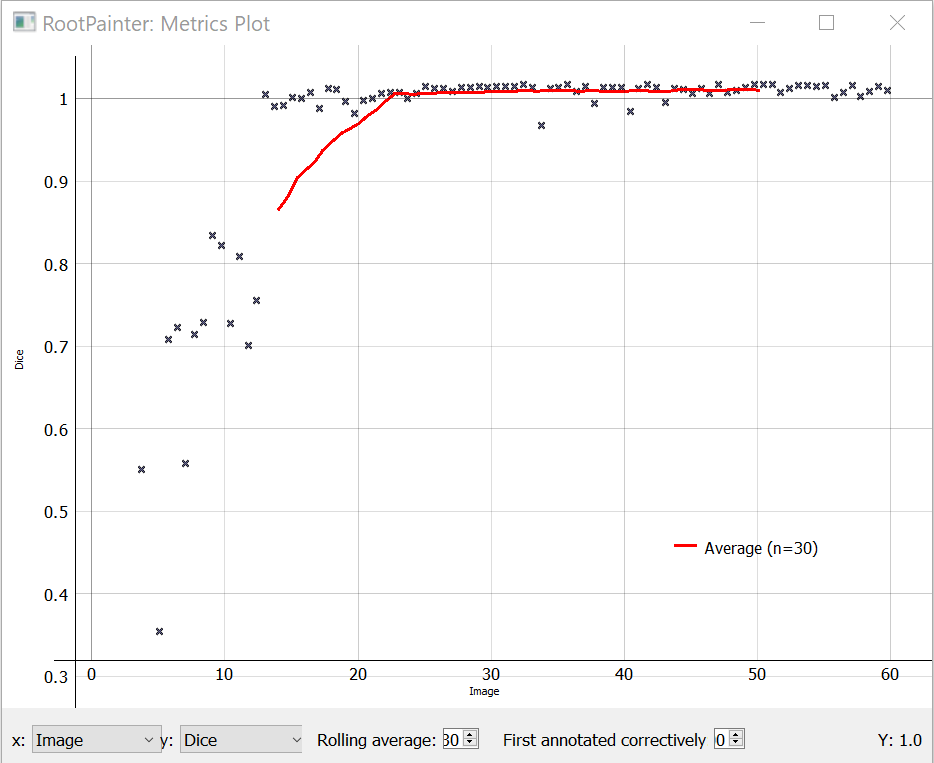


B

**Fig. S4.** *Dice score during root segmentation model development with RootPainter. The Dice score, ranging from 0 to 1, indicates the agreement between model predictions and ground truth, with higher scores signifying better agreement. (A) Dice score during segmentation model development for rhizotron images from the glasshouse. (B) Dice score during segmentation model development for minirhizotron images from the field. The trend shows continuous improvement as more images are annotated. Training was stopped when the Dice score approached 1, and the best model was selected.*

# Algorithm Performance and Biological Relevance

**Table S6.** *Internal validation metrics for clustering algorithms.*

| **Algorithm** | **Treatment** | | **Silhouette Score** | **Dunn Index** | **Calinski Harabasz Index** | **Davies Bouldin Index** |
| --- | --- | --- | --- | --- | --- | --- |
| KMeans | | T0 | 0.452 | 0.460 | 22.895 | 0.903 |
| AgglomerativeClustering | | T0 | 0.434 | 0.577 | 20.784 | 0.970 |
| GaussianMixture | | T0 | 0.434 | 0.577 | 20.784 | 0.970 |
| SpectralClustering | | T0 | 0.418 | 0.152 | 20.777 | 0.861 |
| Birch | | T0 | 0.410 | 0.976 | 11.518 | 0.738 |
| KMeans | | T1 | 0.492 | 0.698 | 30.710 | 0.707 |
| AgglomerativeClustering | | T1 | 0.508 | 1.067 | 30.317 | 0.637 |
| GaussianMixture | | T1 | 0.508 | 1.067 | 30.317 | 0.637 |
| SpectralClustering | | T1 | 0.508 | 1.067 | 30.317 | 0.637 |
| Birch | | T1 | 0.508 | 1.067 | 30.317 | 0.637 |

**Table S7.** *Relative contributions of algorithms to model performance. Table showing the permutation importance values for each algorithm, key features with their SHAP importance scores, and hypothesised biological relevance.*

| **Algorithm** | **Permutation Importance** | **Key Features** | **Top SHAP Score** | **Hypothesised Biological Relevance** |
| --- | --- | --- | --- | --- |
| FCM | 0.019 | FCM_centre_x | 0.071 | Horizontal position of fuzzy root clusters; Lateral exploration for soil volume coverage |
| OPTICS | 0.013 | OPTICS_density_points | 0.06 | Ordered clusters density; Sequential root organisation for uptake |
| HDBSCAN | 0.012 | HDBSCAN_density_points | 0.071 | Hierarchical density clusters; Adaptive clustering in moisture pockets |
| DBSCAN | 0.009 | DBSCAN_density_points | 0.053 | Root cluster density; Adaptive clustering in moisture pockets |
| SLIC | 0.008 | SLIC_centre_x | 0.026 | Superpixel segmentation; Root cluster density optimisation |
| K-mean | 0.007 | K-mean_centre_x | 0.034 | Globular cluster positioning; Generic lateral root exploration |
| Mean-shift | 0.005 | Mean-shift_centre_x | 0.047 | Mode-seeking clusters; Generic lateral root exploration |
| GMM | 0.003 | GMM_centre_x | 0.037 | Gaussian mixture positioning; Generic lateral root exploration |
| Custom | 0.003 | Custom_centre_x | 0.025 | Density-based selection; Generic lateral root exploration |

**
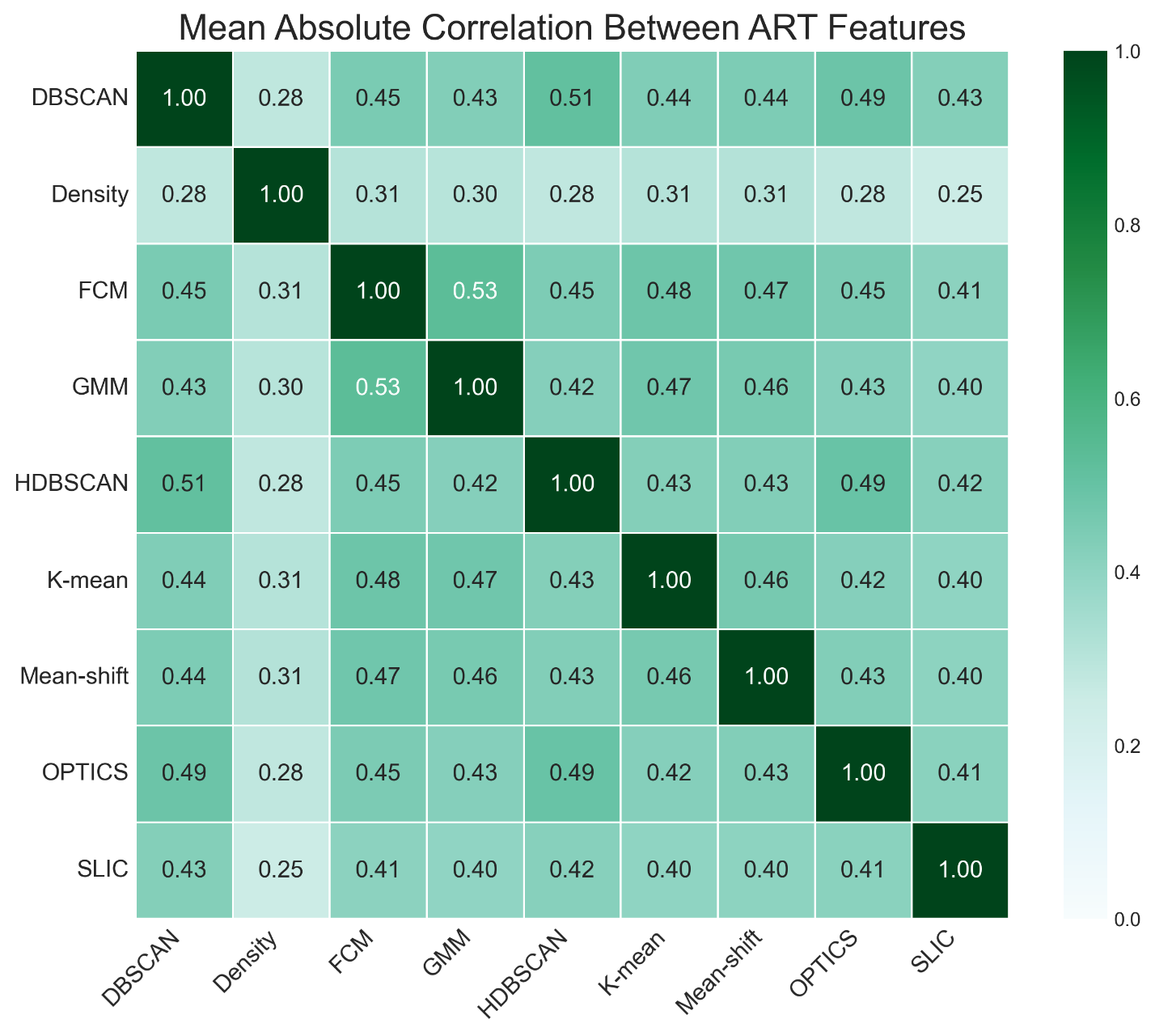
**

**Fig. S5.** *Algorithm feature correlation matrix. Heatmap of the mean absolute Pearson correlation between features generated by different ART algorithm types. Values closer to 0 (lighter colours) indicate less redundancy and more unique information captured by the respective algorithm pairs.*

# Biological Correlations and Trait Stability


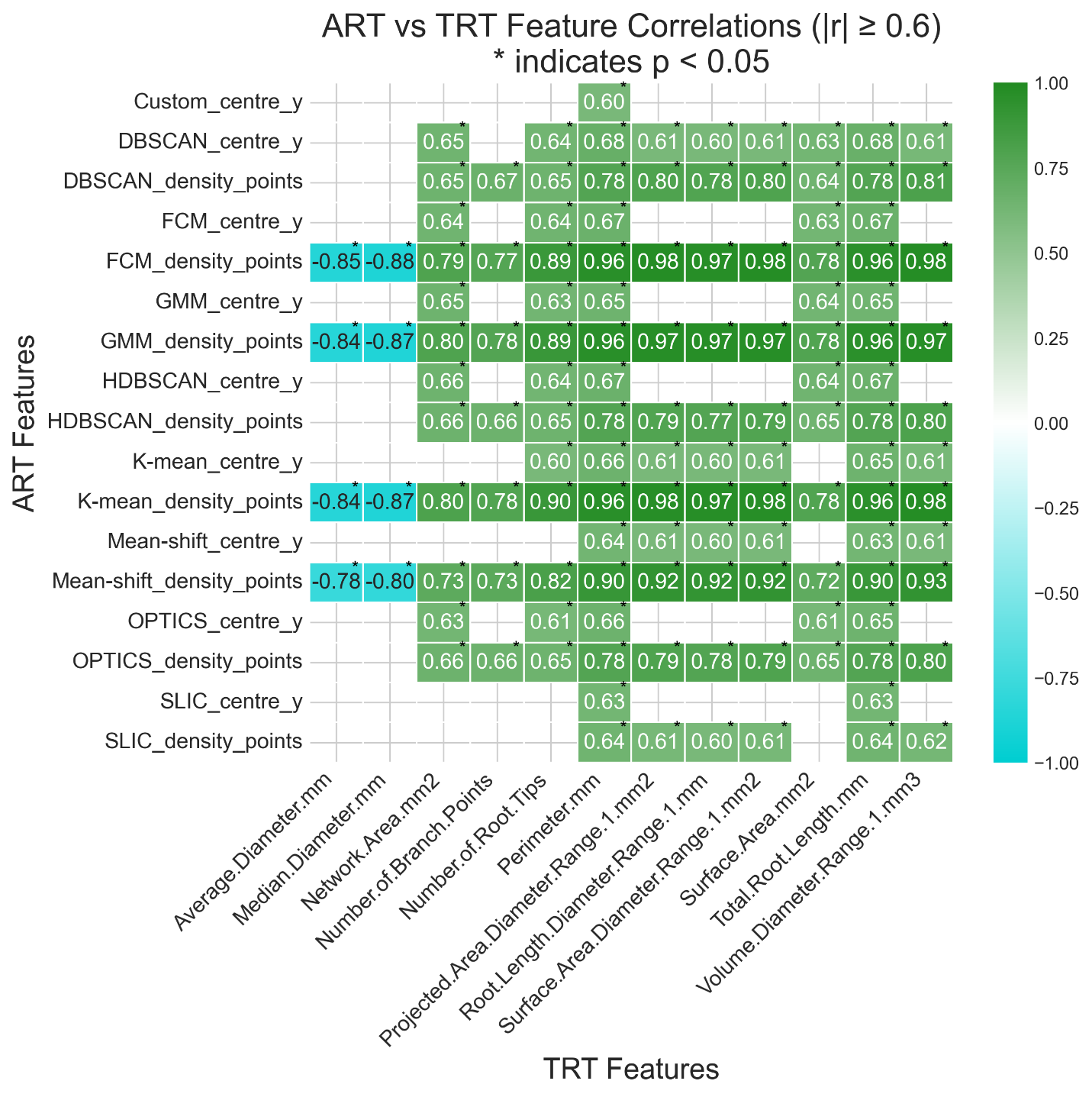


**Fig. S6.** *ART vs TRT Feature Correlations (|r| ≥ 0.6). Heatmap of significant (p < 0.05, indicated by asterisk) Pearson correlations between ART features and TRT features where |r| ≥ 0.6, showing a broader range of biologically meaningful associations.*


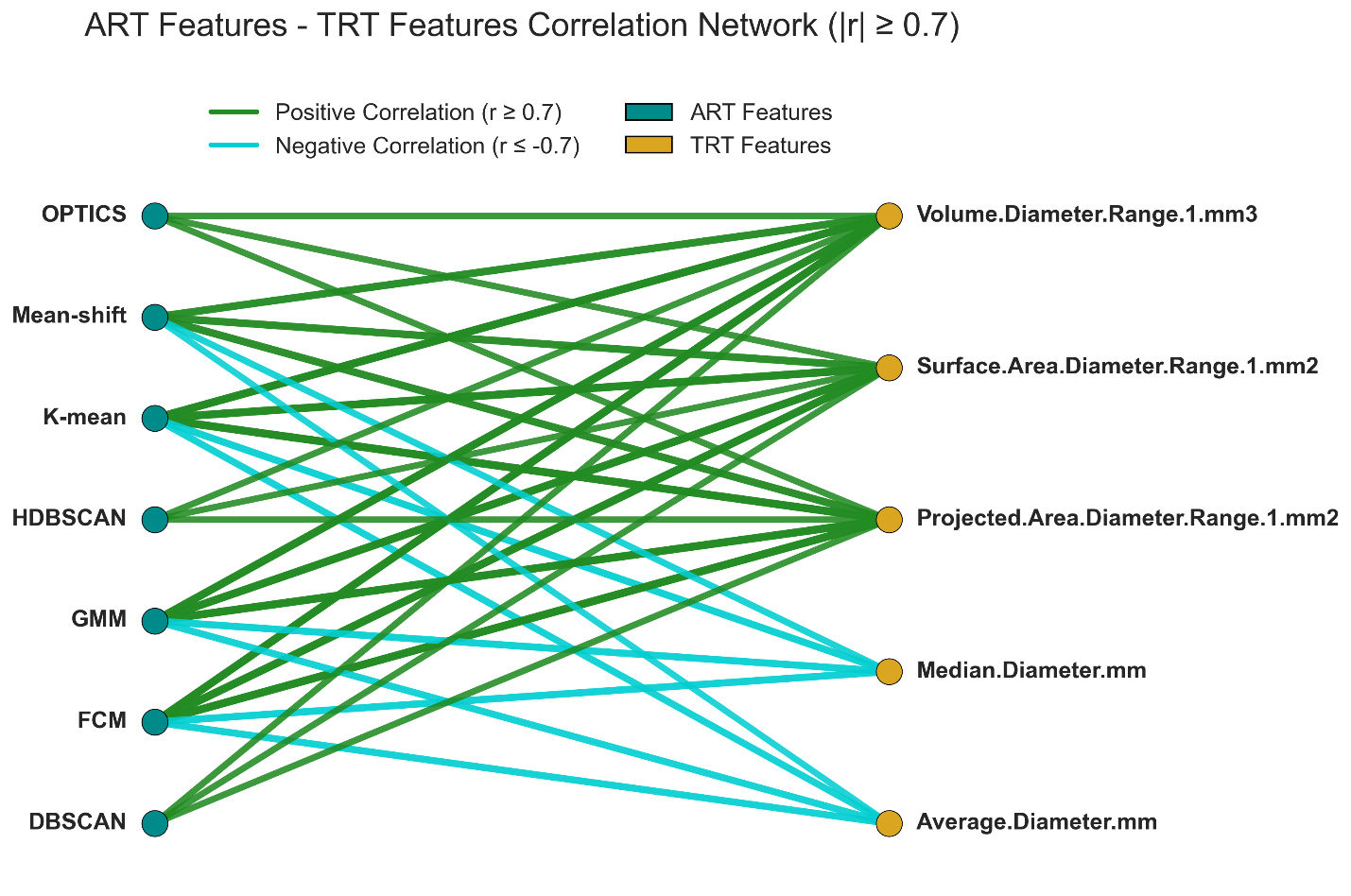


**Fig. S7.** *ART - TRT Feature correlation network. Correlation network visualising significant (|r| ≥ 0.7, or |r| ≥ 0.6 if no 0.7 links exist for an algorithm) relationships between ART families (cyan nodes) and TRT features (gold nodes). Green lines denote positive correlations, and cyan lines denote negative correlations. Line thickness is proportional to the absolute correlation strength.*


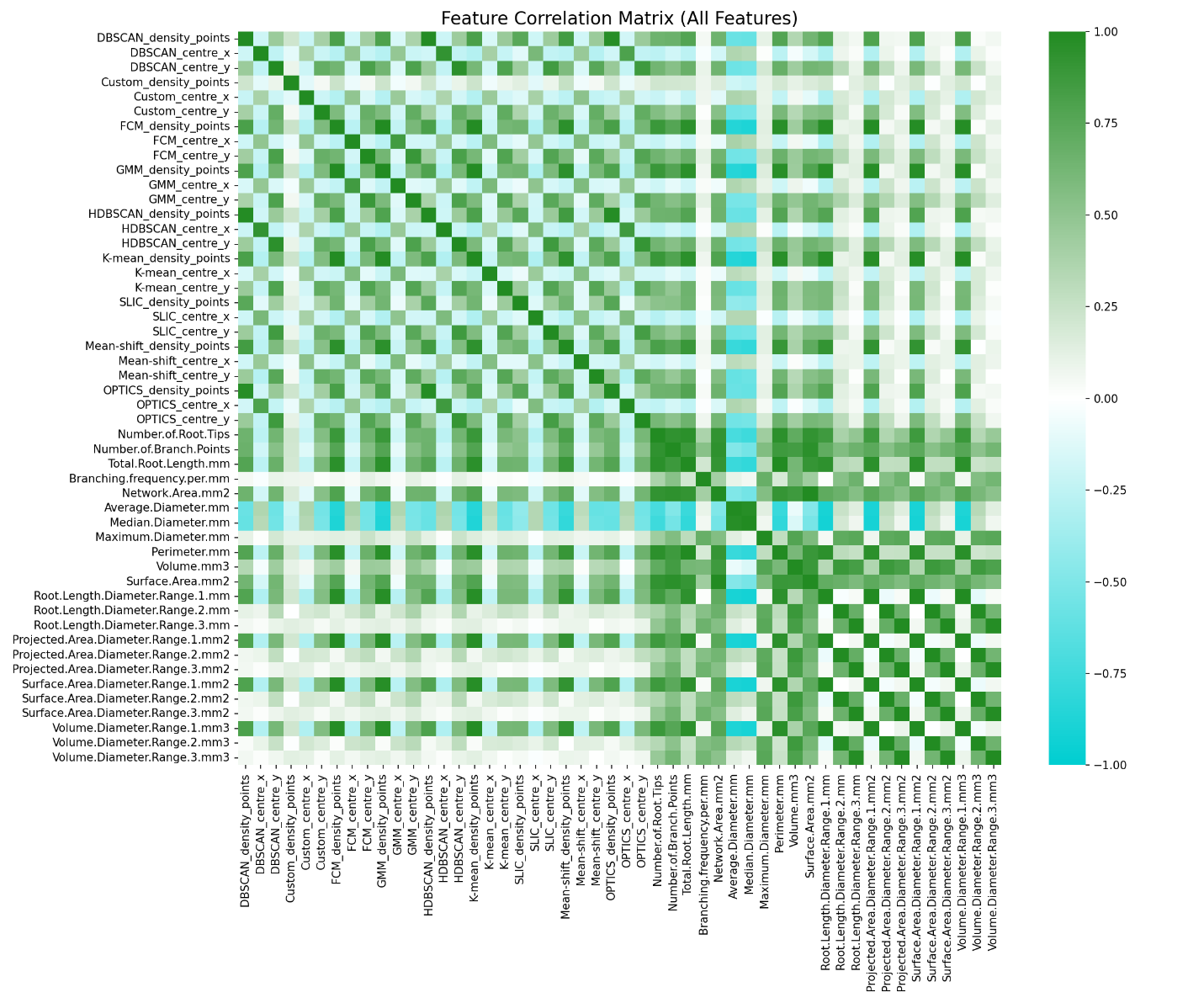


**Fig. S8.** *Feature correlation matrix description: heatmap showing correlations between all features (ART and TRT).*


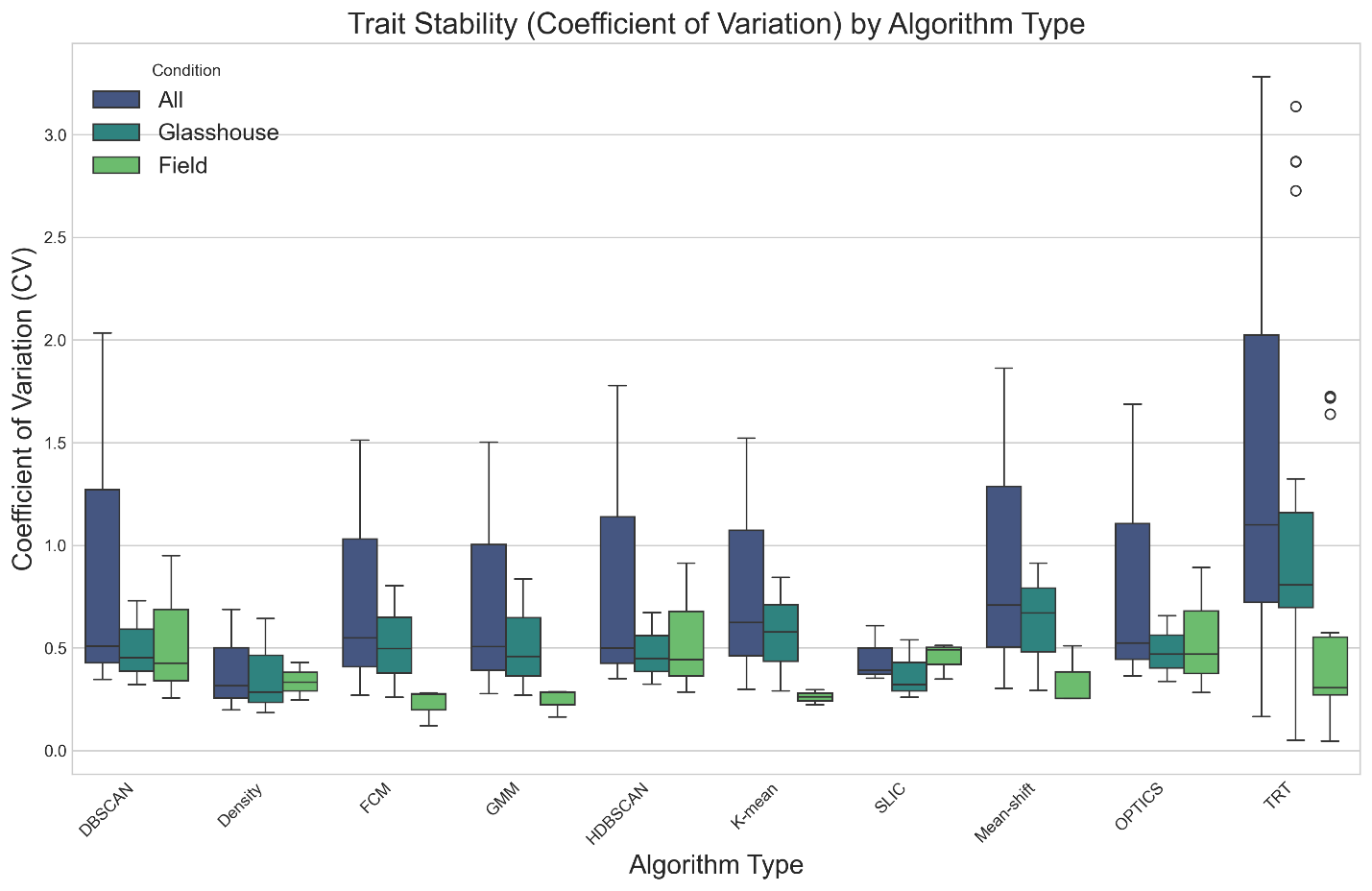


**Fig. S9.** *This boxplot illustrates the Coefficient of Variation (CV) for all 'Algorithm Types' and a combine 'TRT' category for traditional root traits). The data is further categorised by 'Condition' (All, Glasshouse, Field), as indicated by the different coloured boxplots for each algorithm type. The plot aims to compare the trait stability (where a lower CV indicates higher stability or lower relative variability) of features derived from these different algorithms and traditional methods across the specified condition.*

# Model Development and Validation

***
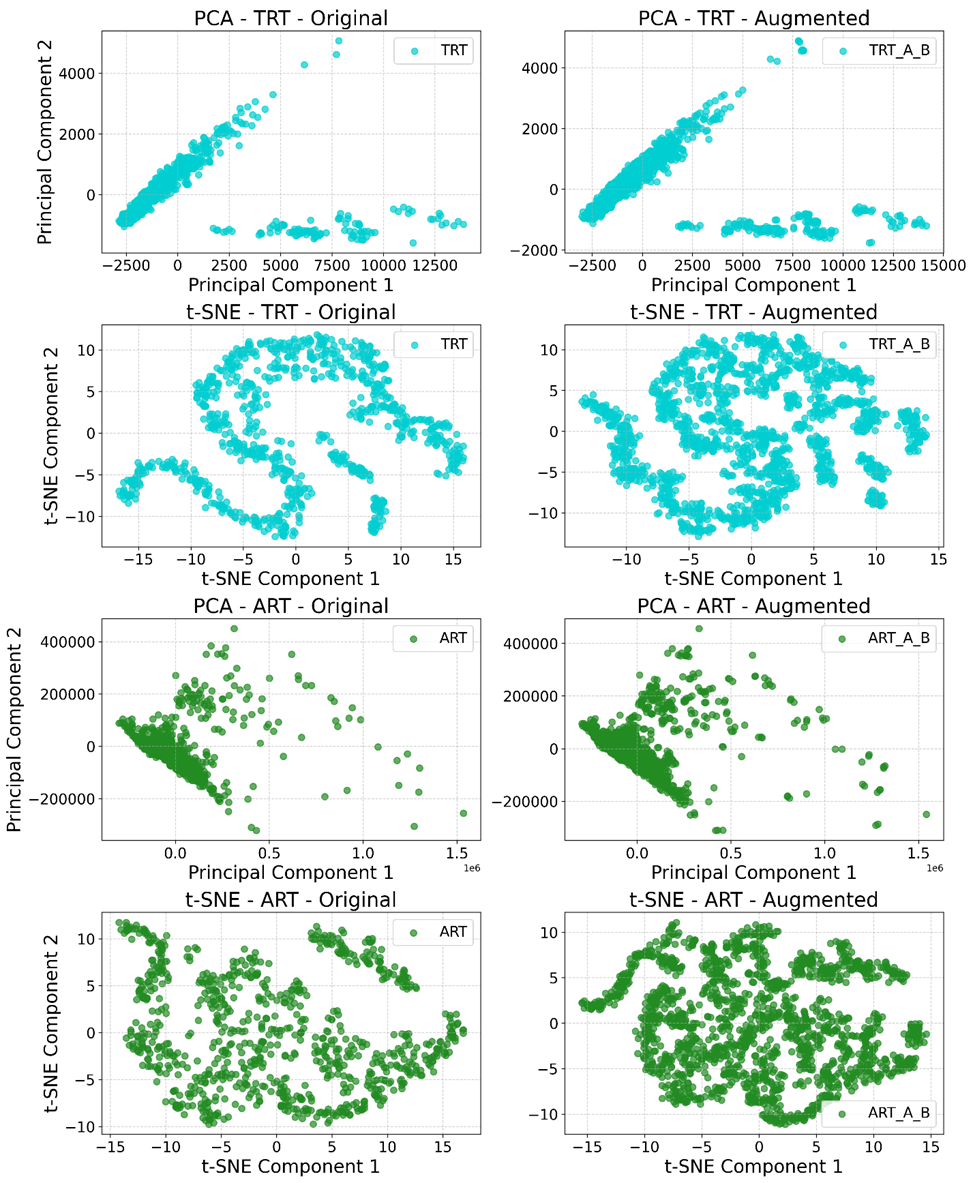
***

**Fig. S10.** *Comparison of original and augmented datasets using PCA and t-SNE plots. (A) and (B) PCA plot of TRT and augmented and bootstrapped data (TRT_A_B). (C) and (D) PCA plot of Combine and augmented and bootstrapped data (Combine_A_B). (E) and (F) t-SNE plot of TRT and augmented and bootstrapped data (TRT_A_B). (G) and (H) t-SNE plot of Combine and augmented and bootstrapped data (Combine_A_B). Both PCA and t-SNE plots demonstrate increased distribution diversity in augmented datasets.*


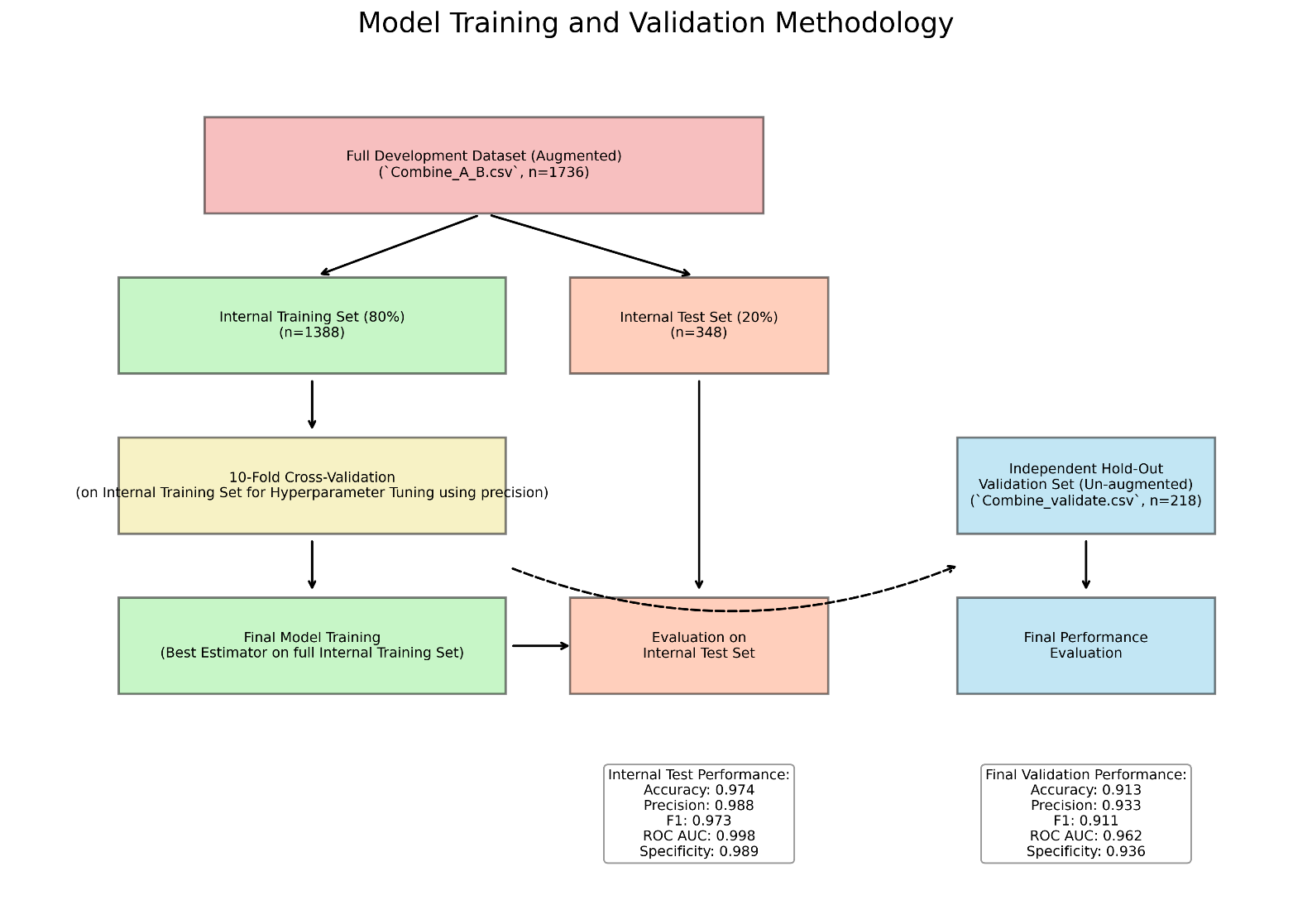


**Fig. S11.** *Validation methodology diagram. Schematic overview of the model training and validation methodology. The full augmented development dataset (n = 1736) was split into an internal training set (80%, n = 1388) and an internal test set (20%, n = 348). Hyperparameters for the Random Forest model was tuned using 10-fold cross-validation on the internal training set (optimising for precision). The final model was trained on the entire internal training set and evaluated on both the internal test set and a completely independent, un-augmented hold-out validation set (n = 218). Key performance metrics are displayed for both evaluation stages.*


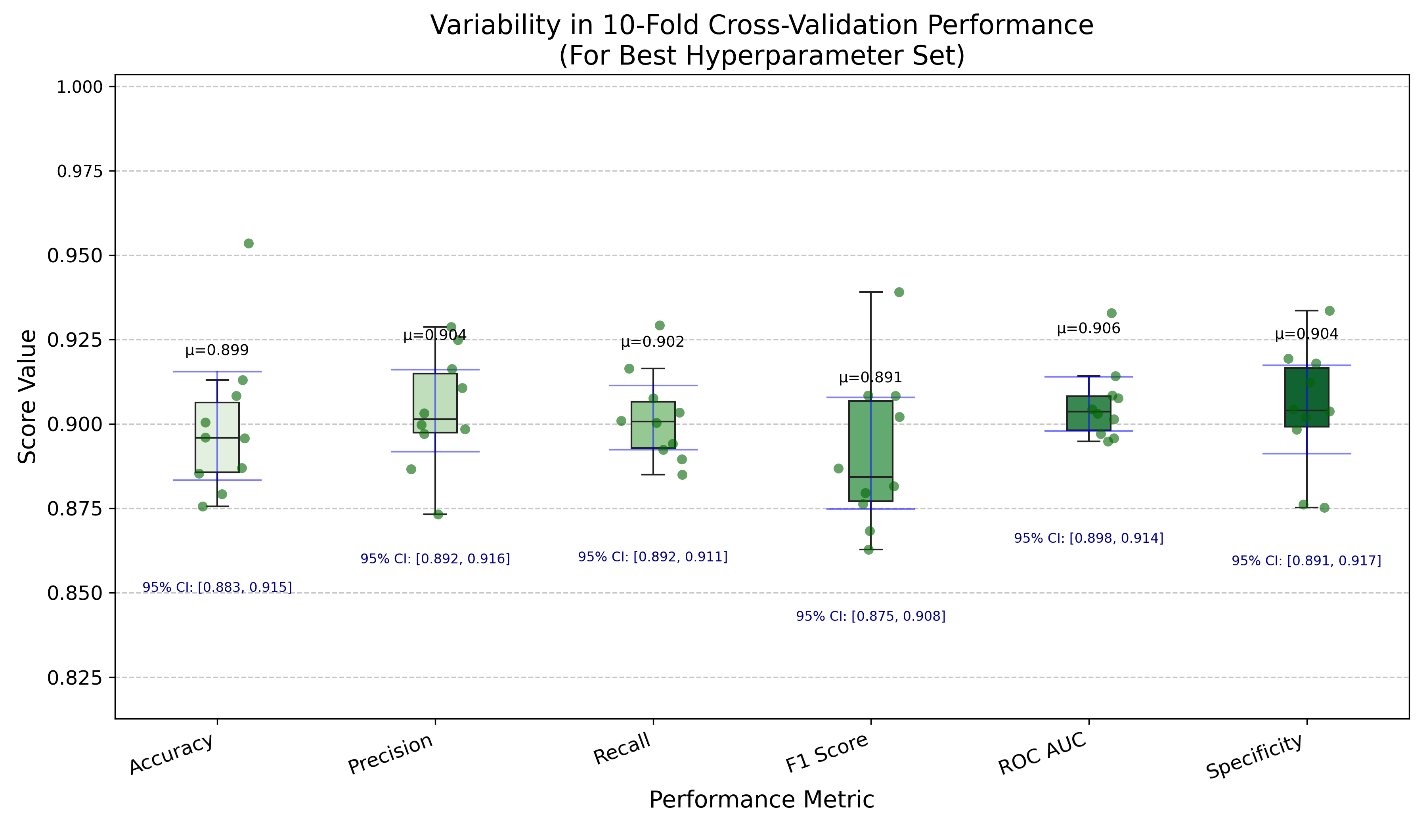


**Fig. S12.** *Cross-validation stability analysis. Variability in 10-fold cross-validation performance for the best hyperparameter set (determined by optimising for precision). Boxplots illustrate the distribution of key performance metrics (Accuracy, Precision, Recall, F1 Score, ROC AUC, and Specificity) across the 10 folds of cross-validation performed on the internal training set. For each metric, the mean value (μ) and 95% confidence interval (CI) are shown. Individual points represent scores from each fold. The low variability (average CV% = 1.95%) demonstrates the model's stability across different data partitions.*

**Table S8.** *Cross-Validation stability metrics. Quantitative assessment of model stability across the 10-fold cross-validation for the best hyperparameter set. For each performance metric, the table provides the mean value, standard deviation (Std), coefficient of variation (CV%), and 95% confidence interval width (CI_Width). The consistently low CV% values (1.242%-2.591%) indicate excellent stability regardless of data partitioning, with precision (our optimization target) showing particularly high consistency (CV% = 1.876%).*

| **Metric** | **Mean** | **Std** | **CV(%)** | **CI_Lower** | **CI_Upper** | **CI_Width** | **N** |
| --- | --- | --- | --- | --- | --- | --- | --- |
| Accuracy | 0.899406 | 0.022474 | 2.498809 | 0.883329 | 0.915483 | 0.032154 | 10 |
| Precision | 0.903876 | 0.016955 | 1.87583 | 0.891747 | 0.916005 | 0.024258 | 10 |
| Recall | 0.901885 | 0.013251 | 1.469292 | 0.892405 | 0.911364 | 0.018959 | 10 |
| F1 Score | 0.891315 | 0.023091 | 2.590689 | 0.874797 | 0.907834 | 0.033037 | 10 |
| ROC AUC | 0.905936 | 0.01125 | 1.241834 | 0.897888 | 0.913984 | 0.016096 | 10 |
| Specificity | 0.904276 | 0.018284 | 2.021932 | 0.891196 | 0.917355 | 0.026159 | 10 |

**
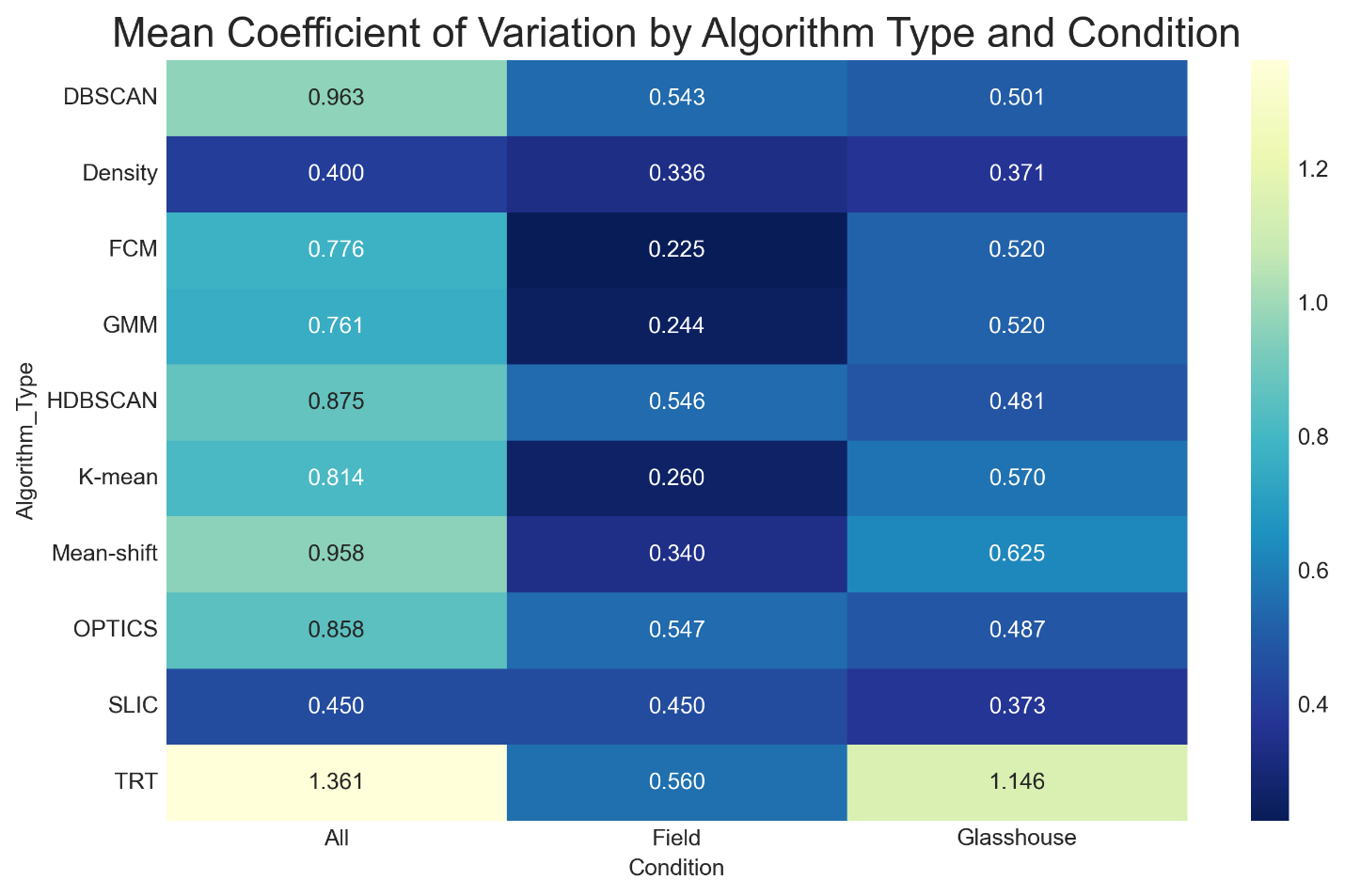
**

**Fig. S13.** *Mean CV by algorithm type and condition Description: Heatmap of mean coefficient of variation by algorithm type and experimental condition, identifying algorithms with best cross-environment stability.*

# Feature Importance and Model Performance


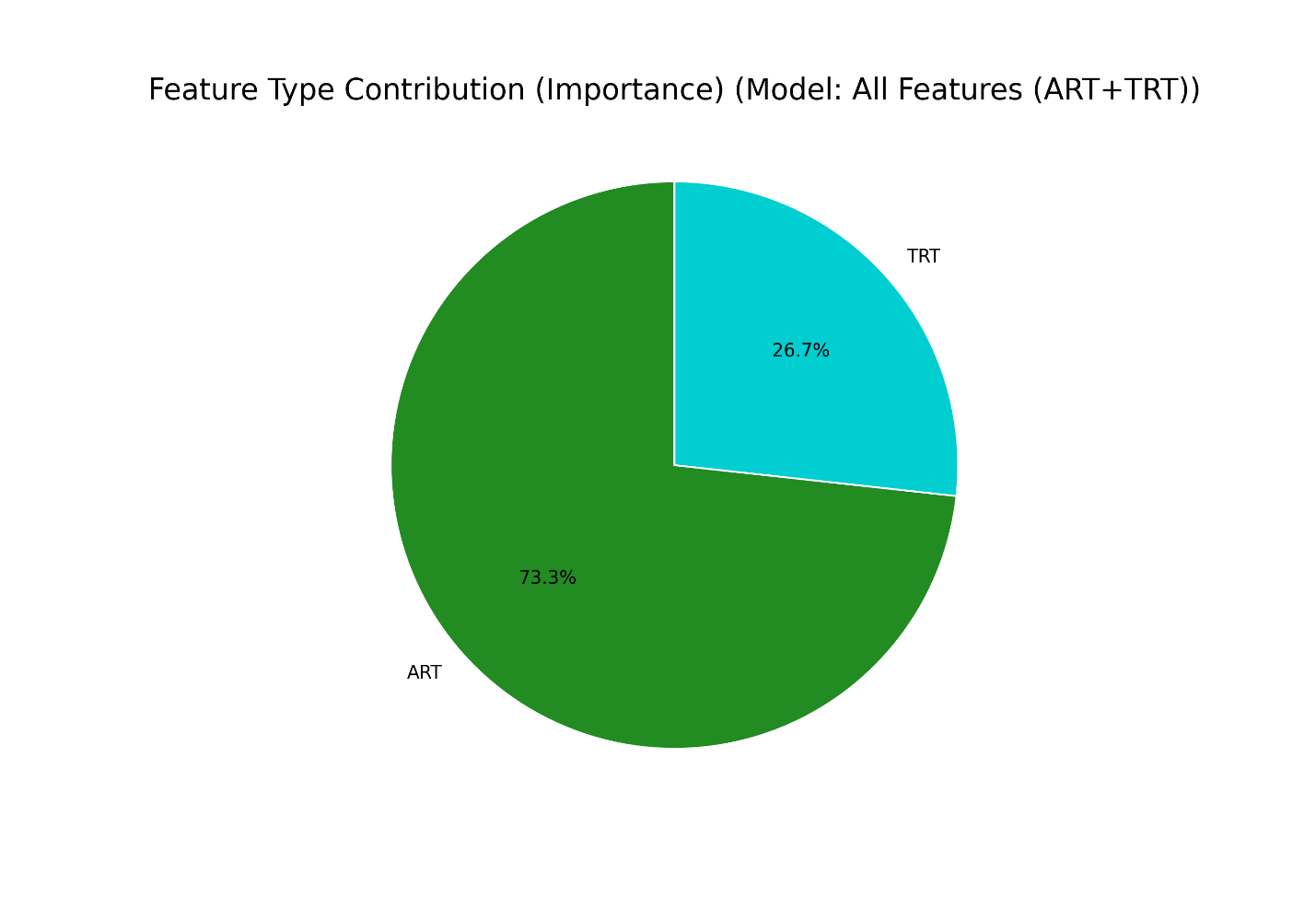


**Fig. S14.** *Feature type contribution. Relative contribution of Algorithmic Root Traits (ARTs) and Traditional Root Traits (TRTs) to model performance based on mean absolute SHAP values from the 'All Features (ART+TRT)' model. Despite representing only 54% of the total features, ARTs contribute 73.3% of the predictive power.*


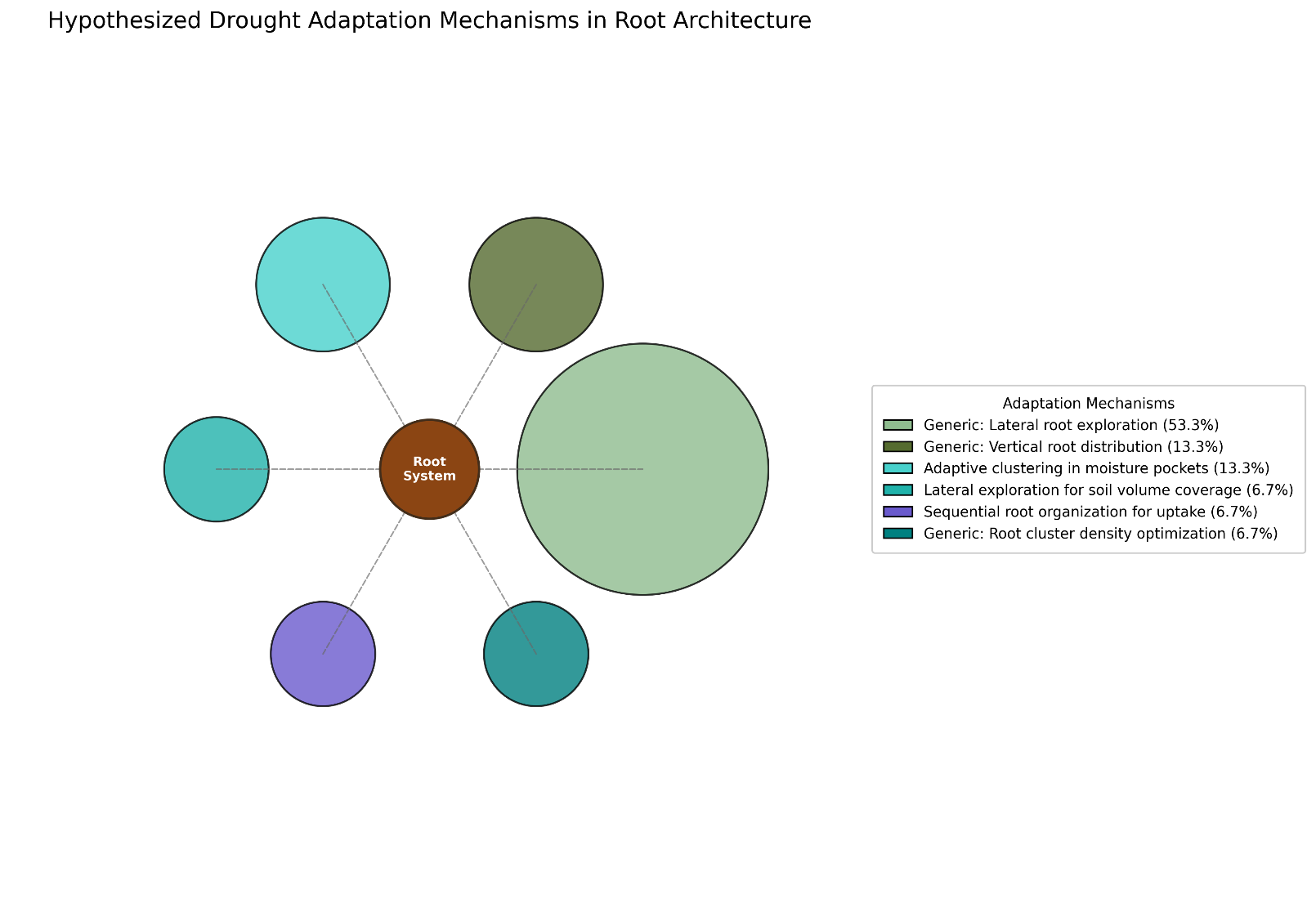


**Fig. S15.** *Hypothesised drought adaptation mechanisms. Conceptual representation of the drought adaptation mechanisms captured by the top ART features from the 'All Features (ART+TRT)' model. The diagram illustrates the relative prominence of different adaptive strategies based on the frequency of top ART features associated with each mechanism. Circle size is proportional to this frequency. Key adaptations highlighted in the legend include "Generic: Lateral root exploration" (53.3%), "Generic: Vertical root distribution" (13.3%), and "Adaptive clustering in moisture pockets" (13.3%).*

Note: The "Hypothesised Drought Adaptation Mechanisms" diagram was derived from the Random Forest model trained on the "All Features (ART+TRT)" dataset. The importance of each Algorithmic Root Trait (ART) feature within this model was determined by its mean absolute SHAP (SHapley Additive exPlanations) value. The top 15 ART features, ranked by these SHAP values (see Table S7 for details on these features, their SHAP scores, and their individual hypothesised biological relevance), were selected for this analysis.

Each of these 15 ART features was programmatically assigned a 'Hypothesised Drought Adaptation Mechanism' by the get_drought_mechanism function within the baseline_comparison_shap_6.py script. This function applies a set of predefined rules to interpret patterns in the ART feature names (e.g., _centre_y indicating vertical distribution, _density_points suggesting clustering, or specific feature names mapping to more granular mechanisms).

The frequency of occurrence for these programmatically assigned mechanisms was then tallied across the 15 top ART features. These frequencies were used to calculate the percentage contribution for each distinct mechanism category displayed in the diagram's legend, which may involve grouping similar individual mechanisms. The categories and their contributions, accounting for all 15 top ART features, are:

Generic: Lateral root exploration: 53.3% (representing 8 out of 15 features)

Generic: Vertical root distribution: 13.3% (representing 2 out of 15 features)

Adaptive clustering in moisture pockets: 13.3% (representing 2 out of 15 features)

Lateral exploration for soil volume coverage: 6.7% (representing 1 out of 15 features)

Sequential root organisation for uptake: 6.7% (representing 1 out of 15 features)

Generic: Root cluster density optimisation: 6.7% (representing 1 out of 15 features)

Visually, the diagram connects a central "Root System" node to peripheral circles, each representing one of these mechanism categories. The radius of each mechanism's circle is scaled proportionally to its frequency count (radius ∝ frequency^0.65), thereby illustrating the relative prominence of each hypothesised adaptive strategy among the features most influential to the model.


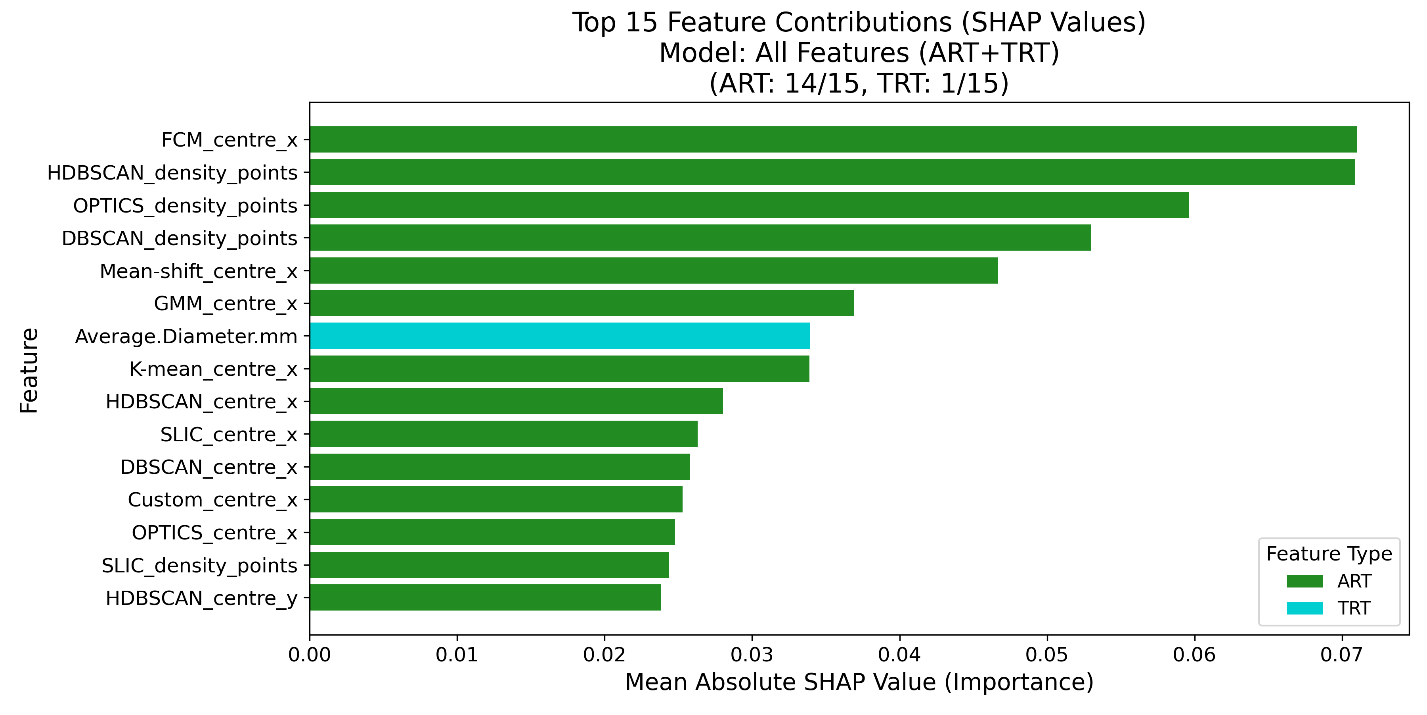


**Fig. S16.** *Top 15 Individual feature contributions. Top 15 most important features from the 'All Features (ART+TRT)' model, ranked by mean absolute SHAP values. ART features dominate the top positions, with FCM_centre_x and HDBSCAN_density_points being the most impactful overall.*


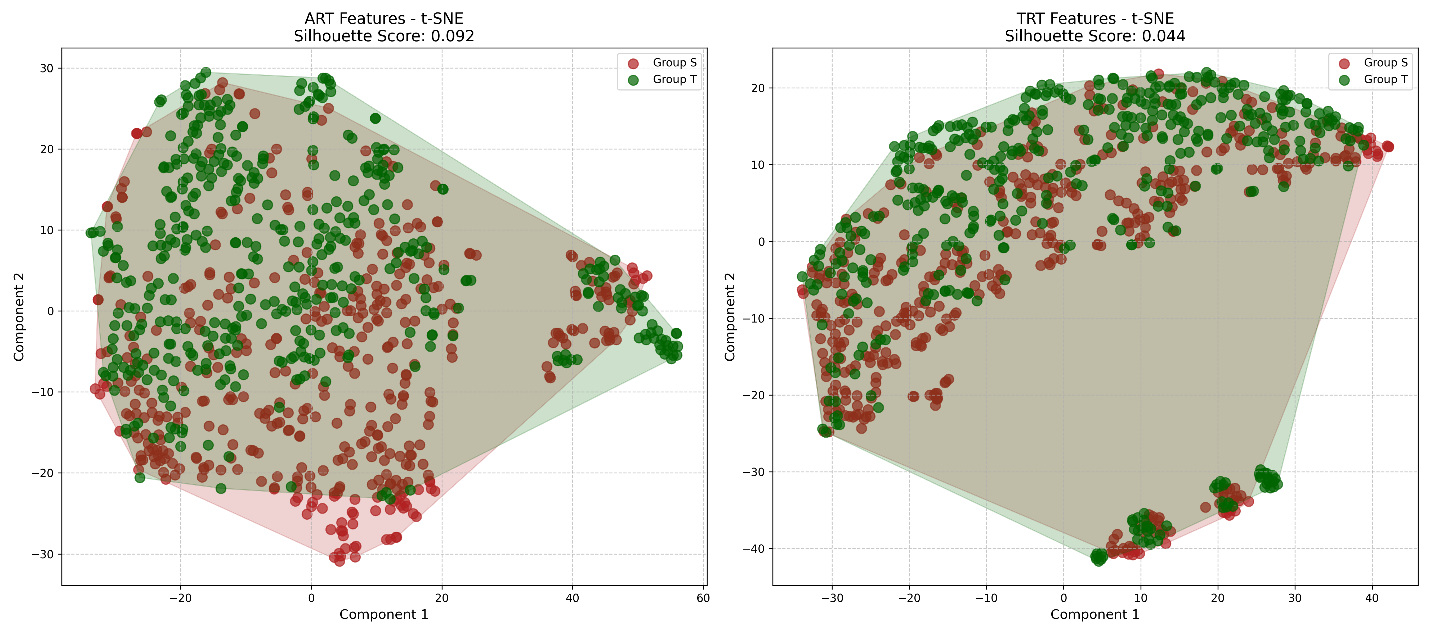


**Fig. S17.** *t-SNE Comparison of ART vs TRT feature spaces. t-SNE projections showing drought-tolerant (green) and drought-susceptible (red) genotypes in (A) ART and (B) TRT feature spaces. Data points include 95% confidence ellipses to highlight group separation. ART features provide substantially superior group separation (silhouette score: 0.092 vs 0.044).*

**Table S9.** *Comprehensive feature importance values across models. This table presents the importance values for all features across different model configurations (All ARTs, All Features (ART+TRT), All TRTs, selected 4 ARTs, Selected 4 TRTs, Selected TRTs+ARTs, and Baseline). This detailed quantitative data supports the feature importance analysis presented in Figures S14 and S16 and demonstrates the consistent dominance of ART features, particularly FCM_centre_x, HDBSCAN_density_points, and OPTICS_density_points across models.*

| **Feature** | **Importance** | **Feature_Type** | **Model** |
| --- | --- | --- | --- |
| FCM_centre_x | 0.081651 | ART | All ARTs |
| DBSCAN_density_points | 0.074981 | ART | All ARTs |
| OPTICS_density_points | 0.073769 | ART | All ARTs |
| HDBSCAN_density_points | 0.072179 | ART | All ARTs |
| Mean-shift_centre_x | 0.053449 | ART | All ARTs |
| GMM_centre_x | 0.052605 | ART | All ARTs |
| K-mean_centre_x | 0.04229 | ART | All ARTs |
| SLIC_centre_x | 0.042076 | ART | All ARTs |
| DBSCAN_centre_y | 0.041266 | ART | All ARTs |
| DBSCAN_centre_x | 0.035813 | ART | All ARTs |
| HDBSCAN_centre_y | 0.034441 | ART | All ARTs |
| OPTICS_centre_x | 0.032205 | ART | All ARTs |
| GMM_centre_y | 0.031736 | ART | All ARTs |
| HDBSCAN_centre_x | 0.03169 | ART | All ARTs |
| FCM_centre_y | 0.030842 | ART | All ARTs |
| OPTICS_centre_y | 0.029639 | ART | All ARTs |
| Custom_centre_x | 0.027288 | ART | All ARTs |
| Mean-shift_centre_y | 0.026375 | ART | All ARTs |
| Mean-shift_density_points | 0.025637 | ART | All ARTs |
| SLIC_density_points | 0.025428 | ART | All ARTs |
| SLIC_centre_y | 0.024351 | ART | All ARTs |
| K-mean_centre_y | 0.021728 | ART | All ARTs |
| K-mean_density_points | 0.020601 | ART | All ARTs |
| FCM_density_points | 0.019961 | ART | All ARTs |
| GMM_density_points | 0.017776 | ART | All ARTs |
| Custom_density_points | 0.016273 | ART | All ARTs |
| Custom_centre_y | 0.013953 | ART | All ARTs |
| FCM_centre_x | 0.070996 | ART | All Features (ART+TRT) |
| HDBSCAN_density_points | 0.07089 | ART | All Features (ART+TRT) |
| OPTICS_density_points | 0.059607 | ART | All Features (ART+TRT) |
| DBSCAN_density_points | 0.052967 | ART | All Features (ART+TRT) |
| Mean-shift_centre_x | 0.04668 | ART | All Features (ART+TRT) |
| GMM_centre_x | 0.036932 | ART | All Features (ART+TRT) |
| Average.Diameter.mm | 0.033912 | TRT | All Features (ART+TRT) |
| K-mean_centre_x | 0.033888 | ART | All Features (ART+TRT) |
| HDBSCAN_centre_x | 0.028036 | ART | All Features (ART+TRT) |
| SLIC_centre_x | 0.026317 | ART | All Features (ART+TRT) |
| DBSCAN_centre_x | 0.025789 | ART | All Features (ART+TRT) |
| Custom_centre_x | 0.025295 | ART | All Features (ART+TRT) |
| OPTICS_centre_x | 0.024782 | ART | All Features (ART+TRT) |
| SLIC_density_points | 0.024365 | ART | All Features (ART+TRT) |
| HDBSCAN_centre_y | 0.023816 | ART | All Features (ART+TRT) |
| Branching.frequency.per.mm | 0.023256 | TRT | All Features (ART+TRT) |
| DBSCAN_centre_y | 0.022083 | ART | All Features (ART+TRT) |
| GMM_centre_y | 0.021627 | ART | All Features (ART+TRT) |
| SLIC_centre_y | 0.018402 | ART | All Features (ART+TRT) |
| FCM_centre_y | 0.015897 | ART | All Features (ART+TRT) |
| K-mean_centre_y | 0.015859 | ART | All Features (ART+TRT) |
| OPTICS_centre_y | 0.015794 | ART | All Features (ART+TRT) |
| Number.of.Root.Tips | 0.015156 | TRT | All Features (ART+TRT) |
| Mean-shift_centre_y | 0.014961 | ART | All Features (ART+TRT) |
| Maximum.Diameter.mm | 0.01281 | TRT | All Features (ART+TRT) |
| Root.Length.Diameter.Range.1.mm | 0.012557 | TRT | All Features (ART+TRT) |
| Volume.Diameter.Range.3.mm3 | 0.012071 | TRT | All Features (ART+TRT) |
| Surface.Area.Diameter.Range.2.mm2 | 0.011968 | TRT | All Features (ART+TRT) |
| Mean-shift_density_points | 0.011399 | ART | All Features (ART+TRT) |
| Number.of.Branch.Points | 0.010833 | TRT | All Features (ART+TRT) |
| Custom_density_points | 0.010793 | ART | All Features (ART+TRT) |
| Projected.Area.Diameter.Range.2.mm2 | 0.009771 | TRT | All Features (ART+TRT) |
| Surface.Area.Diameter.Range.1.mm2 | 0.009741 | TRT | All Features (ART+TRT) |
| FCM_density_points | 0.009667 | ART | All Features (ART+TRT) |
| Projected.Area.Diameter.Range.1.mm2 | 0.009633 | TRT | All Features (ART+TRT) |
| Root.Length.Diameter.Range.2.mm | 0.009402 | TRT | All Features (ART+TRT) |
| Median.Diameter.mm | 0.009311 | TRT | All Features (ART+TRT) |
| K-mean_density_points | 0.0093 | ART | All Features (ART+TRT) |
| Root.Length.Diameter.Range.3.mm | 0.009218 | TRT | All Features (ART+TRT) |
| Network.Area.mm2 | 0.008909 | TRT | All Features (ART+TRT) |
| Volume.Diameter.Range.2.mm3 | 0.008825 | TRT | All Features (ART+TRT) |
| Surface.Area.Diameter.Range.3.mm2 | 0.008762 | TRT | All Features (ART+TRT) |
| Custom_centre_y | 0.008753 | ART | All Features (ART+TRT) |
| Volume.Diameter.Range.1.mm3 | 0.008732 | TRT | All Features (ART+TRT) |
| Perimeter.mm | 0.008694 | TRT | All Features (ART+TRT) |
| Total.Root.Length.mm | 0.00864 | TRT | All Features (ART+TRT) |
| Volume.mm3 | 0.008469 | TRT | All Features (ART+TRT) |
| Surface.Area.mm2 | 0.008429 | TRT | All Features (ART+TRT) |
| Projected.Area.Diameter.Range.3.mm2 | 0.008286 | TRT | All Features (ART+TRT) |
| GMM_density_points | 0.007721 | ART | All Features (ART+TRT) |
| Average.Diameter.mm | 0.088393 | TRT | All TRTs |
| Branching.frequency.per.mm | 0.084057 | TRT | All TRTs |
| Number.of.Root.Tips | 0.06687 | TRT | All TRTs |
| Root.Length.Diameter.Range.2.mm | 0.049926 | TRT | All TRTs |
| Maximum.Diameter.mm | 0.046677 | TRT | All TRTs |
| Volume.mm3 | 0.045621 | TRT | All TRTs |
| Surface.Area.Diameter.Range.2.mm2 | 0.044908 | TRT | All TRTs |
| Network.Area.mm2 | 0.043871 | TRT | All TRTs |
| Number.of.Branch.Points | 0.041907 | TRT | All TRTs |
| Surface.Area.mm2 | 0.041197 | TRT | All TRTs |
| Volume.Diameter.Range.2.mm3 | 0.04109 | TRT | All TRTs |
| Root.Length.Diameter.Range.1.mm | 0.040422 | TRT | All TRTs |
| Projected.Area.Diameter.Range.2.mm2 | 0.04001 | TRT | All TRTs |
| Projected.Area.Diameter.Range.1.mm2 | 0.03916 | TRT | All TRTs |
| Volume.Diameter.Range.1.mm3 | 0.038128 | TRT | All TRTs |
| Projected.Area.Diameter.Range.3.mm2 | 0.035866 | TRT | All TRTs |
| Surface.Area.Diameter.Range.1.mm2 | 0.033092 | TRT | All TRTs |
| Volume.Diameter.Range.3.mm3 | 0.03198 | TRT | All TRTs |
| Perimeter.mm | 0.031304 | TRT | All TRTs |
| Root.Length.Diameter.Range.3.mm | 0.030753 | TRT | All TRTs |
| Total.Root.Length.mm | 0.029774 | TRT | All TRTs |
| Surface.Area.Diameter.Range.3.mm2 | 0.028475 | TRT | All TRTs |
| Median.Diameter.mm | 0.026519 | TRT | All TRTs |
| Total.Root.Length.mm | 1 | TRT | Baseline (Total.Root.Length.mm) |
| FCM_centre_x | 0.34095 | ART | Selected 4 ARTs |
| HDBSCAN_density_points | 0.23341 | ART | Selected 4 ARTs |
| OPTICS_density_points | 0.227308 | ART | Selected 4 ARTs |
| DBSCAN_density_points | 0.198332 | ART | Selected 4 ARTs |
| Volume.mm3 | 0.266735 | TRT | Selected 4 TRTs |
| Total.Root.Length.mm | 0.25701 | TRT | Selected 4 TRTs |
| Network.Area.mm2 | 0.246895 | TRT | Selected 4 TRTs |
| Surface.Area.mm2 | 0.229359 | TRT | Selected 4 TRTs |
| FCM_centre_x | 0.228256 | ART | Selected TRTs+ARTs |
| HDBSCAN_density_points | 0.15278 | ART | Selected TRTs+ARTs |
| OPTICS_density_points | 0.138376 | ART | Selected TRTs+ARTs |
| DBSCAN_density_points | 0.125975 | ART | Selected TRTs+ARTs |
| Volume.mm3 | 0.092278 | TRT | Selected TRTs+ARTs |
| Total.Root.Length.mm | 0.087952 | TRT | Selected TRTs+ARTs |
| Network.Area.mm2 | 0.087207 | TRT | Selected TRTs+ARTs |
| Surface.Area.mm2 | 0.087177 | TRT | Selected TRTs+ARTs |

**Table S10.** *Comparative model performance metrics. This table presents the quantitative performance metrics (Accuracy, Precision, Recall, F1, ROC_AUC, CV_Accuracy_Mean, CV_Accuracy_Std) for each model configuration tested (Baseline, selected 4 TRTs, Selected 4 ARTs, Selected TRTs+ARTs, All TRTs, All ARTs, All Features). The data numerically demonstrates the superior performance of ART-based models and the synergistic effect when combining ARTs with TRTs.*

| **Model** | **Features** | **Accuracy** | **Precision** | **Recall** | **F1** | **ROC_AUC** | **CV_Accuracy_Mean** | **CV_Accuracy_Std** |
| --- | --- | --- | --- | --- | --- | --- | --- | --- |
| **Baseline (Total.Root.Length.mm)** | 1 | 0.577586 | 0.577944 | 0.577828 | 0.577499 | 0.61656 | 0.588145 | 0.022623 |
| **Selected 4 TRTs** | 4 | 0.70977 | 0.70981 | 0.709831 | 0.709768 | 0.76807 | 0.7143 | 0.024365 |
| **Selected 4 ARTs** | 4 | 0.850575 | 0.850575 | 0.850621 | 0.85057 | 0.924931 | 0.861749 | 0.010493 |
| **Selected TRTs+ARTs** | 8 | 0.899425 | 0.900199 | 0.899181 | 0.899325 | 0.961912 | 0.910154 | 0.018699 |
| **All TRTs** | 23 | 0.856322 | 0.860423 | 0.855708 | 0.855745 | 0.926582 | 0.882512 | 0.030886 |
| **All ARTs** | 27 | 0.962644 | 0.962861 | 0.96254 | 0.962629 | 0.996779 | 0.975821 | 0.019646 |
| **All Features (ART+TRT)** | 50 | 0.974138 | 0.974675 | 0.973969 | 0.974121 | 0.997605 | 0.974093 | 0.019076 |
